# Application of 1-D discrete wavelet transform based compressed sensing matrices for speech compression

**DOI:** 10.1186/s40064-016-3740-x

**Published:** 2016-11-30

**Authors:** Yuvraj V. Parkale, Sanjay L. Nalbalwar

**Affiliations:** Department of Electronics and Telecommunication Engineering, Dr. Babasaheb Ambedkar Technological University, Lonere, Maharashtra India

**Keywords:** Speech compression, Compressed sensing (CS), Discrete wavelet transform (DWT), Mean opinion score (MOS), Perceptual evaluation of speech quality (PESQ)

## Abstract

**Background:**

Compressed sensing is a novel signal compression technique in which signal is compressed while sensing. The compressed signal is recovered with the only few numbers of observations compared to conventional Shannon–Nyquist sampling, and thus reduces the storage requirements. In this study, we have proposed the 1-D discrete wavelet transform (DWT) based sensing matrices for speech signal compression. The present study investigates the performance analysis of the different DWT based sensing matrices such as: Daubechies, Coiflets, Symlets, Battle, Beylkin and Vaidyanathan wavelet families.

**Results:**

First, we have proposed the Daubechies wavelet family based sensing matrices. The experimental result indicates that the db10 wavelet based sensing matrix exhibits the better performance compared to other Daubechies wavelet based sensing matrices. Second, we have proposed the Coiflets wavelet family based sensing matrices. The result shows that the coif5 wavelet based sensing matrix exhibits the best performance. Third, we have proposed the sensing matrices based on Symlets wavelet family. The result indicates that the sym9 wavelet based sensing matrix demonstrates the less reconstruction time and the less relative error, and thus exhibits the good performance compared to other Symlets wavelet based sensing matrices. Next, we have proposed the DWT based sensing matrices using the Battle, Beylkin and the Vaidyanathan wavelet families. The Beylkin wavelet based sensing matrix demonstrates the less reconstruction time and relative error, and thus exhibits the good performance compared to the Battle and the Vaidyanathan wavelet based sensing matrices. Further, an attempt was made to find out the best-proposed DWT based sensing matrix, and the result reveals that sym9 wavelet based sensing matrix shows the better performance among all other proposed matrices. Subsequently, the study demonstrates the performance analysis of the sym9 wavelet based sensing matrix and state-of-the-art random and deterministic sensing matrices.

**Conclusions:**

The result reveals that the proposed sym9 wavelet matrix exhibits the better performance compared to state-of-the-art sensing matrices. Finally, speech quality is evaluated using the MOS, PESQ and the information based measures. The test result confirms that the proposed sym9 wavelet based sensing matrix shows the better MOS and PESQ score indicating the good quality of speech.

## Introduction

Conventional signal processing methods such as Fourier transform and a short time Fourier transform (STFT) are inadequate for the analysis of non-stationary signals which have abrupt transitions superimposed on the lower frequency backgrounds such as the speech, music and bio-electric signals. The wavelet transform (WT) (Daubechie Ingrid 1992) overcomes these drawbacks and provides both the time resolution and frequency resolution of a signal. The basic idea of the wavelet transform is to represent the signal to be analyzed as a superposition of wavelets. The wavelet transform is the most popular signal analysis tool, and it is successfully used in different application areas such as speech or audio and image compression.

**Fig. 1:**
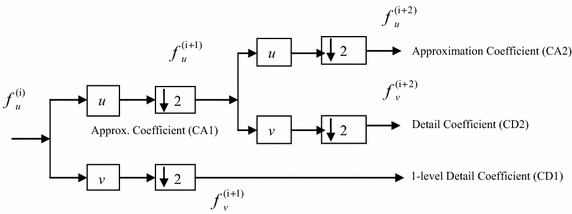
Block diagram of 1-D fast forward wavelet transform with 2-level decomposition

Given an input signal *x* of length *N*, the wavelet transform consists of log_2_ *N* decomposition levels. The input signal decomposition is accomplished through a series filtering and downsampling processes. The reconstruction of the original signal is accomplished through an upsampling, series filtering and adding all the sub-bands. Figure 1 shows the block diagram of 1-D forward wavelet transform with 2-level decomposition (Mallat 2009; Meyer 1993). The input signal is filtered using the low-pass filter (*u*) and the high-pass filter (*v*). A filtering is achieved by computing a linear convolution between the input signal and the filter coefficients. The two filters are chosen such that, they are orthogonal to each other and provides a perfect reconstruction of the original signal *x*. Therefore, the quadrature mirror filter (QMF) is commonly used for the perfect reconstruction of a two-channel filter bank.

Wavelet analysis provides approximation coefficients and detail coefficients. The low frequency information about the signal is given by the approximation, while the high frequency information is given by the detail coefficients. Since the low frequency signal is of more importance than the high frequency signal, the output of the low-pass filter is used as an input for the next decomposition stages; whereas the output of high-pass filter is used at the time of signal reconstruction. The wavelet coefficients are computed by using a series filtering and downsampling processes. The wavelet coefficients (*f*) are given by:

1 $$f = {\mathbf{W}}x$$

where **W** is the *N* × *N* wavelet matrix and defined as: **W** = **WI**, where **I** is *N* × *N* identity matrix.

Thus, the classical approach of data compression is to employ the discrete wavelet transform (DWT) based methods (Skodras and Ebrahimi 2001) prior to the transmission. However, these methods includes the complicated multiplications, exhaustive coefficient search and sorting procedure along with the arithmetic encoding of the significant coefficients with their locations, which consequently results in a huge storage requirement and power consumption. Furthermore, the smooth oscillatory signals such as the speech or music signals will be compressed more efficiently in the wavelet packet basis compared to the wavelet representation. Coifman and Wickerhauser (1992) proposed the algorithm for an efficient data compression based on the Shannon entropy for the best basis selection. The orthogonal wavelet packets and localized trigonometric functions are exploited as a basis. This allows an efficient compression of a voice and image signals; however, at the cost of an additional computation in searching the best wavelet packet basis.

The research work presented on CS by Donoho (2006), Baraniuk (2007), Candes and Wakin (2008), and Donoho and Tsaig (2006) have energized the research in many application areas like medical image processing (Lustig et al. 2008), wireless sensor networks (Guan et al. 2011), analog-to-information converters (AIC) (Laska et al. 2007), communications and networks (Berger et al. 2010), radar (Qu and Yang 2012), etc.

In the paper Liu et al. (2014) successfully implemented the CS based compression and the wavelet based compression procedure on the field programmable gate array (FPGA). The result shows that the CS based procedure achieves the better performance compared to the wavelet compression in terms of power consumption and the number of computing resources required. Furthermore, the sparse binary sensing matrix achieves the desired signal compression, but at the price of the higher signal reconstruction time and the higher sensing matrix construction time.

Candes et al. (2006a, b) proposed an i.i.d. (independent identical distribution) Gaussian or Bernoulli random sensing matrices for the compressed sensing. However, the practical implementation of these sensing matrices requires the huge computational cost and memory storage requirements, and therefore considered as inappropriate for large scale applications.

Rauhut (2009), Haupt et al. (2010), Xu et al. (2014), Yin et al. (2010), and Sebert et al. (2008) exploited the Toeplitz and Circulant sensing matrices which effectively recover the original signal with the reduction in the computational cost and the memory requirement.

As an alternative to the random sensing matrices, the authors in Arash and Farokh (2011) proposed the deterministic construction of sensing matrices such as binary, bipolar and the ternary matrices. Several authors have proposed the deterministic construction of sensing matrices using the codes such as the sparse binary matrices based on the low density parity check (LDPC) code (Lu and Kpalma 2012), chirp sensing codes (Applebauma et al. 2009), scrambled block Hadamard matrices (Gan et al. 2008), Reed–Muller sensing codes (Howard et al. 2008) and the Vandermond matrices (DeVore 2007).

The restricted isometry property (RIP) is just a sufficient condition for an exact signal recovery. Even though, the deterministic sensing matrices are an incapable to satisfy RIP condition, they are very useful in practice because of the deterministic nature of the sampler and might be able to advance some features like compression ratio and computational complexity.

The successful implementation of the CS technique is depends on the efficient design of the sensing matrices which are used to compress the given signal. Since, the DWT shows a very good energy compaction property, it can be used for designing the sensing matrices. In this study, we have proposed the 1-D discrete wavelet transform (DWT) based sensing matrices for speech signal compression. The major contributions of the research paper are the proposed 1-D DWT sensing matrices based on different wavelet families such as the Daubechies, Coiflets, Symlets, Battle, Beylkin and the Vaidyanathan wavelet families. Furthermore, the proposed DWT based sensing matrices are compared with state-of-the-art random and the deterministic sensing matrices. Besides, the speech quality is evaluated using mean opinion sore (MOS) and the perceptual evaluation of speech quality (PESQ) measures.

The paper is organized as follows. Section two briefly introduces the compressed sensing (CS) theory with signal acquisition and reconstruction model. Section three describes the proposed methodology for the discrete wavelet transform (DWT) matrix. Experimental results and discussion are presented in section four. Finally, section five presents the conclusions.

## Compressed sensing (CS) framework

### Background

Compressed sensing is a novel signal compression technique in which signal is acquired and compressed simultaneously. The signal is recovered with the only few number of observations compared to the conventional Shannon–Nyquist sampling which requires observations that are twice the signal bandwidth. Compressed sensing is performed with two basic steps: signal acquisition and signal reconstruction.

### CS signal acquisition model

Compressed sensing technique is illustrated as follows:

2 $$y = {\varvec{\Phi}}f$$

where *f* is the input signal of length *N* × 1, *y* is the compressed output signal of length *M* × 1, and **Φ** is *M* × *N* sensing matrix.

The input signal *f* is sparse in some sparsifying domain (**Ψ**) and given as:

3 $$f = {\varvec{\Psi}}x$$

where *x* is the non-sparse input signal. Combined form of Eqs. (2) and (3) is given as:

4 $$y = {\varvec{\Theta}}f = {\mathbf{\varPhi \varPsi }}x$$

The two basic conditions should be satisfied for the successful implementation of the CS.

1. Sensing matrix (**Φ**) and sparsity transform (**Ψ**) should be incoherent to each other.
2. The **Φ** should satisfy the restricted isometric property (RIP) (Candes and Tao 2006) and defined as follow:
  5 $$(1 - \mathop \delta \nolimits_{k} )\mathop {\left\| x \right\|}\nolimits_{2}^{2} \le \mathop {\left\| {{\varvec{\Phi}}x} \right\|}\nolimits_{2}^{2} \le (1 + \mathop \delta \nolimits_{k} )\mathop {\left\| x \right\|}\nolimits_{2}^{2}$$
  where *δ*_*k*_ ∊ (0, 1) is called as restricted isometric constant of the matrix and *k* is the number of non-zero coefficients.

### CS signal reconstruction model

Since, the compressed sensing technique use only a few number of observations, there are large number of solutions. Therefore, the different optimization based algorithms are used to find the exact sparse solution. The basic algorithms are based on the norm minimization such as L0-norm, L1-norm and L2-norm. Out of these three, L1-norm is widely used, because of its ability to recover the exact sparse solution along with the efficient reconstruction speed. Presently, there are different recovery algorithm available such as the basis pursuit (BP) (Chen et al. 2001), orthogonal matching pursuit (OMP) (Tropp and Gilbert 2007), etc.

## The proposed 1-D discrete wavelet transform (DWT) matrix

### 1-D DWT matrix

For a signal *x* of length *N* = 2^*n*^ and a low-pass filter (*u*), the *i*th level wavelet decomposition (Vidakovic 1999; Wang and Vieira 2010) is given by an Eqs. (6) and (7). Where, *v* is the high-pass filter.

6 $$\mathop f\nolimits_{u}^{(i)} (j) = \sum\limits_{k = 1}^{{\mathop 2\nolimits^{n - i + 1} }} {u(k - 2j)\mathop f\nolimits_{u}^{(i - 1)} } (k)\quad {\text{where,}}\quad j = 1,2, \ldots ,\mathop 2\nolimits^{n - i}$$

And

7 $$\mathop f\nolimits_{v}^{(i)} (j) = \sum\limits_{k = 1}^{{\mathop 2\nolimits^{n - i + 1} }} {v(k - 2j)\mathop f\nolimits_{u}^{(i - 1)} } (k)\quad {\text{where}},\quad j = 1,2, \ldots ,\mathop 2\nolimits^{n - i}$$

The reconstruction of $f_{u}^{i - 1}$ from *f*_*u*_^*i*^ and *f*_*v*_^*i*^ can be obtained by

8 $$\mathop f\nolimits_{u}^{(i - 1)} (j) = \sum\limits_{k = 1}^{{\mathop 2\nolimits^{n - i} }} {u(j - 2k)} \mathop f\nolimits_{u}^{(i)} (k) + \sum\limits_{k = 1}^{{\mathop 2\nolimits^{n - i} }} {v(j - 2k)} \mathop f\nolimits_{v}^{(i)} (k)$$

The 1-D DWT matrix forms are given as below:

9 $$\mathop f\nolimits_{u}^{(i)} = \mathop U\nolimits^{(i)} \mathop f\nolimits_{u}^{(i - 1)}$$

and

10 $$\mathop f\nolimits_{v}^{(i)} = \mathop V\nolimits^{(i)} \mathop f\nolimits_{v}^{(i - 1)}$$

where, $f_{u}^{(i)}$ is the 2^*n*−*i*^ dimensional low pass vector in the *i*th level and $f_{v}^{(i)}$ the high-pass, while $f_{u}^{(i - 1)}$ is the 2^*n*−*i*+1^ dimensional low-pass vector in the (*i* − 1)th level. The two 2^*n*−*i*^ by 2^*n*−*i*+1^ wavelet filter matrices are given below.

11 $$\mathop U\nolimits^{(i)} = \left[ {\begin{array}{*{20}c} {u( - 1)} & 0 & 0 & {\begin{array}{*{20}c} 0 & \cdots & {u( - 3)} & {u( - 2)} \\ \end{array} } \\ {u( - 3)} & {u( - 2)} & {u( - 1)} & {\begin{array}{*{20}c} 0 & \cdots & {u( - 5)} & {u( - 4)} \\ \end{array} } \\ \vdots & \vdots & \vdots & {\begin{array}{*{20}c} \vdots & \ddots & \vdots & \vdots \\ \end{array} } \\ 0 & 0 & 0 & {\begin{array}{*{20}c} 0 & \cdots & {u( - 1)} & 0 \\ \end{array} } \\ \end{array} } \right]$$

And

12 $$\mathop V\nolimits^{(i)} = \left[ {\begin{array}{*{20}c} {v( - 1)} & 0 & 0 & {\begin{array}{*{20}c} 0 & \cdots & {v( - 3)} & {v( - 2)} \\ \end{array} } \\ {v( - 3)} & {v( - 2)} & {v( - 1)} & {\begin{array}{*{20}c} 0 & \cdots & {v( - 5)} & {v( - 4)} \\ \end{array} } \\ \vdots & \vdots & \vdots & {\begin{array}{*{20}c} \vdots & \ddots & \vdots & \vdots \\ \end{array} } \\ 0 & 0 & 0 & {\begin{array}{*{20}c} 0 & \cdots & {v( - 1)} & 0 \\ \end{array} } \\ \end{array} } \right]$$

Thus, the *i*th scale wavelet transform can be represented as:

13 $$\left[ {\begin{array}{*{20}c} {\mathop f\nolimits_{u}^{(i)} } \\ {\mathop f\nolimits_{v}^{(i)} } \\ \end{array} } \right] = \left[ {\begin{array}{*{20}c} {\mathop U\nolimits^{(i)} } \\ {\mathop V\nolimits^{(i)} } \\ \end{array} } \right]\mathop f\nolimits_{u}^{(i - 1)}$$

This gives the wavelet matrix of 1-level decomposition. The wavelet matrix for different levels of decomposition is given as below.

14 $$\mathop f\nolimits_{u}^{(i - 1)} = \mathop U\nolimits^{(i - 1)} \mathop f\nolimits_{u}^{(i - 2)}$$

Above equation can be represented as,

15 $$\left[ {\begin{array}{*{20}c} {\mathop f\nolimits_{u}^{(i)} } \\ {\mathop f\nolimits_{v}^{(i)} } \\ {\mathop f\nolimits_{v}^{(i - 1)} } \\ {\begin{array}{*{20}c} \vdots \\ {\mathop f\nolimits_{v}^{(2)} } \\ {\mathop f\nolimits_{v}^{(1)} } \\ \end{array} } \\ \end{array} } \right] = \left[ {\begin{array}{*{20}c} {\mathop U\nolimits^{(i)} \mathop U\nolimits^{(i - 1)} \cdots \mathop U\nolimits^{(1)} } \\ {\mathop V\nolimits^{(i)} \mathop U\nolimits^{(i - 1)} \cdots \mathop U\nolimits^{(1)} } \\ {\mathop V\nolimits^{(i)} \mathop U\nolimits^{(i - 2)} \cdots \mathop U\nolimits^{(1)} } \\ {\begin{array}{*{20}c} \vdots \\ {\mathop V\nolimits^{(2)} \mathop U\nolimits^{(1)} } \\ {\mathop V\nolimits^{(1)} } \\ \end{array} } \\ \end{array} } \right]x$$

Here, the numbers of signal decomposition levels are restricted to 2^*n*−*i*+1^ ≥ *L*. Where, *L* is the length of the filter.

Thus, the final wavelet transform matrix is given by an Eq. (16).

16 $${\mathbf{W}} = \left[ {\begin{array}{*{20}c} {\mathop U\nolimits^{(i)} \mathop U\nolimits^{(i - 1)} \cdots \mathop U\nolimits^{(1)} } \\ {\mathop V\nolimits^{(i)} \mathop U\nolimits^{(i - 1)} \cdots \mathop U\nolimits^{(1)} } \\ {\mathop V\nolimits^{(i)} \mathop U\nolimits^{(i - 2)} \cdots \mathop U\nolimits^{(1)} } \\ {\begin{array}{*{20}c} \vdots \\ {\mathop V\nolimits^{(2)} \mathop U\nolimits^{(1)} } \\ {\mathop V\nolimits^{(1)} } \\ \end{array} } \\ \end{array} } \right]$$

### Design procedure for the proposed 1-D DWT based sensing matrices

Following are the procedural steps to construct 1-D DWT based sensing matrices.

1. Create a desired quadrature mirror filters (QMF) such as Daubechies, Coiflets, Symlets, Beylkin, Vaidyanathan and Battle filters. For example db1 (Haar) filter is given as *f* = [1 1] and the db2 filter is formed as follows:
  17 $$f = \left[ {\begin{array}{*{20}c} {0.482962913145} & {0.836516303738} \\ {0.224143868042} & { - 0.129409522551} \\ \end{array} } \right]$$
2. Create the *N* × *N* Identity matrix.
3. Perform 1-D forward wavelet transform on the *N* × *N* Identity matrix. Thus, the *N* × *N* wavelet transform matrix is generated.
4. Select the first *m* number of rows to form the *m* × *N* DWT sensing matrix. Where, *m* is the minimum number of measurements.

## Experimental results and discussion

### Methodology

The proposed work is evaluated on the CMU/CSTR KDT US English TIMIT database for speech synthesis by Carnegie Mellon University and Edinburgh University (Edinburgh 2002). The details of the database used are as follows: File name: Kdt_001.wav, channel: 1(Mono), bit rate: 256 kbps, audio sample rate: 16 kHz, total duration: 3 s. The number of samples (*N*) selected are 2048 and the total duration of analyzed speech signal is 0.128 s for simulation. The experimental work is performed using MATLAB 7.8.0 (R2009a) software with Intel (R) CORE 2 Duo CPU, 3 GB RAM system specifications. The discrete cosine transform (DCT) is used as the sparsifying basis for speech signal because of its high sparsity. The speech compression is performed using the sensing matrices based on the different DWT families (Donoho et al. 2007). The basis pursuit (BP) (Chen et al. 2001) is used as signal recovery algorithm for speech signal.

The performance of the reconstructed speech signal is evaluated using the metrics like compression ratio (CR), root mean square error (RMSE), relative error, signal to noise ratio (SNR), signal reconstruction time and sensing matrix construction time.

CR is obtained using relation,

18 $$CR = \frac{M}{N}$$

where *N* is the length of speech signal and *M is* the number of measurements taken from sensing matrix.

RMSE is given as below:

19 $${\text{RMSE}} = \sqrt {\frac{{\sum\nolimits_{n = 1}^{N} {\mathop {(x(n) - \tilde{x}(n))}\nolimits^{2} } }}{N}}$$

where *x*(*n*) is the original signal and $\tilde{x}(n)$ is the reconstructed signal.

Relative error is defined as:

20 $$Rel.Error = \frac{{\left\| {\tilde{x}(n) - x(n)} \right\|_{2} }}{{\left\| {x(n)} \right\|_{2} }}$$

where *x*(*n*) is the original signal and $\tilde{x}(n)$ is the reconstructed signal.

SNR is obtained as,

21 $$SNR(db) = 20\log \left( {\frac{{\left\| {x(n)} \right\|_{2} }}{{\left\| {x(n) - \tilde{x}(n)} \right\|_{2} }}} \right)$$

where *x*(*n*) is the original signal and $\tilde{x}(n)$ is the reconstructed signal.

Besides, signal reconstruction time is computed to provide the amount of time required to recover the original signal using reconstruction algorithm. The amount of time required to construct the sensing matrix is also an important parameter and should be minimum.

### Performance analysis of the Daubechies wavelet family based sensing matrices

This section demonstrates the performance analysis of the different DWT sensing matrices based on Daubechies wavelet family such as db1, db2, db3, db4, db5, db6, db7, db8, db9, db10. The speech signal of length 2048 is taken with 50% sparsity level, preserving the only 1024 number of non-zeros. For a different number of measurements (*m*), corresponding compression ratios (CR), signal reconstruction time (s), relative error, root mean square error (RMSE) and signal-to-noise ratio (SNR) are calculated (Tables 1, 2, 3, 4, 5, 6, 7, 8, 9, 10).

**Table 1:** Performance analysis of the proposed db1 (Haar) wavelet based sensing matrix

Length of signal (*N*)	Number of measurements (*m*)	Compression ratio (CR = *m/N)*	Sparsity level = (*k/N*) × 100 (%)	No. of non-zeros (*k*)	No. of iterations required	Signal reconstruction time (s)	RMSE	Relative error	SNR (db)	Construction time for sensing matrix (s)
2048	205	0.1	50	1024	16	1.281382	0.0585	0.9774	0.1985	2.584058
2048	410	0.2	50	1024	16	2.331942	0.0521	0.8711	1.1984	2.523152
2048	512	0.25	50	1024	16	3.752369	0.0521	0.8707	1.2024	2.514315
2048	614	0.3	50	1024	16	4.242975	0.0488	0.8153	1.7740	2.570737
2048	849	0.4	50	1024	14	5.853003	0.0479	0.7997	1.9418	2.581421
2048	1024	0.5	50	1024	14	9.486588	0.0478	0.7993	1.9454	2.581856
2048	1229	0.6	50	1024	14	11.080416	0.0471	0.7877	2.0723	2.593704
2048	1434	0.7	50	1024	13	14.440796	0.0468	0.7824	2.1310	2.546346
2048	1536	0.75	50	1024	12	17.799127	0.0468	0.7816	2.1406	2.523620
2048	1638	0.8	50	1024	11	16.568279	0.0468	0.7816	2.1406	2.484885
2048	1843	0.9	50	1024	11	20.629636	0.0468	0.7816	2.1407	2.518902
2048	2048	1.0	50	1024	9	22.525032	0.0468	0.7815	2.1409	2.612855

**Table 2:** Performance analysis of the proposed db2 wavelet based sensing matrix

Length of signal (*N*)	Number of measurements (*m*)	Compression ratio (CR = *m/N)*	Sparsity level = (*k/N*) × 100 (%)	No. of non-zeros (*k*)	No. of iterations required	Signal reconstruction time (s)	RMSE	Relative error	SNR (db)	Construction time for sensing matrix (s)
2048	205	0.1	50	1024	15	1.336137	0.0596	0.9955	0.0391	2.159816
2048	410	0.2	50	1024	15	2.444694	0.0552	0.9224	0.7021	2.187808
2048	512	0.25	50	1024	15	3.622582	0.0552	0.9222	0.7034	2.103215
2048	614	0.3	50	1024	14	3.919803	0.0517	0.8635	1.2751	2.337719
2048	849	0.4	50	1024	13	5.740394	0.0502	0.8387	1.5278	2.105989
2048	1024	0.5	50	1024	12	8.508652	0.0501	0.8376	1.5391	2.319550
2048	1229	0.6	50	1024	12	10.622007	0.0475	0.7931	2.0137	2.055366
2048	1434	0.7	50	1024	12	13.716035	0.0469	0.7838	2.1160	2.378571
2048	1536	0.75	50	1024	11	15.991534	0.0468	0.7817	2.1395	2.135815
2048	1638	0.8	50	1024	11	17.085937	0.0468	0.7816	2.1404	2.078314
2048	1843	0.9	50	1024	11	27.217349	0.0468	0.7816	2.1405	2.423662
2048	2048	1.0	50	1024	9	29.832311	0.0468	0.7815	2.1409	2.365919

**Table 3:** Performance analysis of the proposed db3 wavelet based sensing matrix

Length of signal (*N*)	Number of measurements (*m*)	Compression ratio (CR = *m/N*)	Sparsity level = (*k/N*) × 100 (%)	No. of non-zeros (*k*)	No. of iterations required	Signal reconstruction time (s)	RMSE	Relative error	SNR (db)	Construction time for sensing matrix (s)
2048	205	0.1	50	1024	18	1.626908	0.0567	0.9483	0.4615	2.253346
2048	410	0.2	50	1024	15	2.434635	0.0545	0.9106	0.8137	2.160987
2048	512	0.25	50	1024	15	3.636753	0.0545	0.9099	0.8199	2.252568
2048	614	0.3	50	1024	15	4.033450	0.0502	0.8383	1.5323	2.201634
2048	849	0.4	50	1024	13	5.929769	0.0496	0.8282	1.6373	2.285777
2048	1024	0.5	50	1024	13	9.180948	0.0496	0.8282	1.6372	2.309093
2048	1229	0.6	50	1024	13	12.480212	0.0477	0.7963	1.9788	2.427516
2048	1434	0.7	50	1024	13	16.554802	0.0469	0.7829	2.1258	2.509123
2048	1536	0.75	50	1024	12	27.233861	0.0468	0.7817	2.1396	2.175306
2048	1638	0.8	50	1024	11	16.821100	0.0468	0.7816	2.1406	2.283585
2048	1843	0.9	50	1024	11	28.673976	0.0468	0.7816	2.1407	2.192527
2048	2048	1.0	50	1024	9	30.905552	0.0468	0.7815	2.1409	2.262969

**Table 4:** Performance analysis of the proposed db4 wavelet based sensing matrix

Length of signal (*N*)	Number of measurements (*m*)	Compression ratio (CR = *m/N*)	Sparsity level = (*k/N*) × 100 (%)	No. of non-zeros (*k*)	No. of iterations required	Signal reconstruction time (s)	RMSE	Relative error	SNR (db)	Construction time for sensing matrix (s)
2048	205	0.1	50	1024	15	2.301267	0.0545	0.9099	0.8198	2.260742
2048	410	0.2	50	1024	15	2.332355	0.0524	0.8749	1.1607	2.507263
2048	512	0.25	50	1024	15	3.696636	0.0523	0.8744	1.1657	2.370597
2048	614	0.3	50	1024	15	4.308034	0.0496	0.8283	1.6363	2.161184
2048	849	0.4	50	1024	13	5.856042	0.0484	0.8092	1.8387	2.199477
2048	1024	0.5	50	1024	13	19.062773	0.0484	0.8093	1.8376	2.219947
2048	1229	0.6	50	1024	13	11.001844	0.0472	0.7887	2.0622	2.080746
2048	1434	0.7	50	1024	12	13.359806	0.0468	0.7823	2.1325	2.134800
2048	1536	0.75	50	1024	12	16.748983	0.0468	0.7816	2.1405	2.337097
2048	1638	0.8	50	1024	11	16.197748	0.0468	0.7816	2.1407	2.025083
2048	1843	0.9	50	1024	11	20.308890	0.0468	0.7816	2.1407	2.074552
2048	2048	1.0	50	1024	9	21.916145	0.0468	0.7815	2.1409	2.149653

**Table 5:** Performance analysis of the proposed db5 wavelet based sensing matrix

Length of signal (*N*)	Number of measurements (*m*)	Compression ratio (CR = *m/N*)	Sparsity level = (*k/N*) × 100 (%)	No. of non-zeros (*k*)	No. of iterations required	Signal reconstruction time (s)	RMSE	Relative error	SNR (db)	Construction time for sensing matrix (s)
2048	205	0.1	50	1024	15	1.308421	0.0550	0.9192	0.7316	2.240430
2048	410	0.2	50	1024	15	2.164346	0.0508	0.8496	1.4162	2.211380
2048	512	0.25	50	1024	15	3.399650	0.0509	0.8498	1.4138	2.250745
2048	614	0.3	50	1024	15	3.924939	0.0491	0.8201	1.7221	2.412286
2048	849	0.4	50	1024	14	5.935914	0.0481	0.8030	1.9057	2.270019
2048	1024	0.5	50	1024	14	9.353480	0.0481	0.8031	1.9048	2.337383
2048	1229	0.6	50	1024	13	11.118477	0.0470	0.7848	2.1053	2.216672
2048	1434	0.7	50	1024	13	17.975014	0.0468	0.7822	2.1335	2.245930
2048	1536	0.75	50	1024	11	15.538426	0.0468	0.7816	2.1405	2.440602
2048	1638	0.8	50	1024	11	16.539879	0.0468	0.7816	2.1407	2.182187
2048	1843	0.9	50	1024	11	20.442859	0.0468	0.7816	2.1407	2.235392
2048	2048	1.0	50	1024	9	22.641664	0.0468	0.7815	2.1409	2.210355

**Table 6:** Performance analysis of the proposed db6 wavelet based sensing matrix

Length of signal (*N*)	Number of measurements (*m*)	Compression ratio (CR = *m/N*)	Sparsity level = (*k/N*) × 100 (%)	No. of non-zeros (*k*)	No. of iterations required	Signal reconstruction time (s)	RMSE	Relative error	SNR (db)	Construction time for sensing matrix (s)
2048	205	0.1	50	1024	15	1.257052	0.0554	0.9260	0.6677	2.291140
2048	410	0.2	50	1024	15	2.232353	0.0506	0.8458	1.4544	2.356109
2048	512	0.25	50	1024	16	3.710359	0.0506	0.8454	1.4584	2.357269
2048	614	0.3	50	1024	15	4.001119	0.0494	0.8259	1.6617	2.306622
2048	849	0.4	50	1024	15	6.425953	0.0487	0.8142	1.7855	2.367848
2048	1024	0.5	50	1024	14	9.85939	0.0487	0.8138	1.7893	2.389422
2048	1229	0.6	50	1024	13	15.540625	0.0469	0.7845	2.1080	2.698509
2048	1434	0.7	50	1024	13	18.172868	0.0468	0.7827	2.1286	2.627686
2048	1536	0.75	50	1024	12	21.322045	0.0468	0.7816	2.1404	2.699761
2048	1638	0.8	50	1024	12	30.749301	0.0468	0.7816	2.1406	2.608611
2048	1843	0.9	50	1024	12	39.662306	0.0468	0.7816	2.1406	2.706883
2048	2048	1.0	50	1024	9	37.150618	0.0468	0.7815	2.1409	2.581647

**Table 7:** Performance analysis of the proposed db7 wavelet based sensing matrix

Length of signal (*N*)	Number of measurements (*m*)	Compression ratio (CR = *m/N*)	Sparsity level = (*k/N*) × 100 (%)	No. of non-zeros (*k*)	No. of iterations required	Signal reconstruction time (s)	RMSE	Relative error	SNR (db)	Construction time for sensing matrix (s)
2048	205	0.1	50	1024	16	1.544091	0.0564	0.9417	0.5216	2.557882
2048	410	0.2	50	1024	16	2.310240	0.0514	0.8584	1.3260	2.503921
2048	512	0.25	50	1024	16	3.655857	0.0514	0.8585	1.3247	2.371664
2048	614	0.3	50	1024	15	4.050385	0.0511	0.8537	1.3739	2.391605
2048	849	0.4	50	1024	14	5.772598	0.0501	0.8379	1.5362	2.502802
2048	1024	0.5	50	1024	14	9.536010	0.0501	0.8375	1.5399	2.450485
2048	1229	0.6	50	1024	12	10.281689	0.0470	0.7848	2.1049	2.458048
2048	1434	0.7	50	1024	12	13.554986	0.0468	0.7827	2.1278	2.505254
2048	1536	0.75	50	1024	12	17.109362	0.0468	0.7816	2.1404	2.616728
2048	1638	0.8	50	1024	12	18.134841	0.0468	0.7816	2.1406	2.543920
2048	1843	0.9	50	1024	11	21.115266	0.0468	0.7816	2.1406	2.512582
2048	2048	1.0	50	1024	9	22.753802	0.0468	0.7815	2.1409	2.492282

**Table 8:** Performance analysis of the proposed db8 wavelet based sensing matrix

Length of signal (*N*)	Number of measurements (*m*)	Compression ratio (CR = *m/N*)	Sparsity level = (*k/N*) × 100 (%)	No. of non-zeros (*k*)	No. of iterations required	Signal reconstruction time (s)	RMSE	Relative error	SNR (db)	Construction time for sensing matrix (s)
2048	205	0.1	50	1024	16	1.379793	0.0562	0.9397	0.5406	2.445416
2048	410	0.2	50	1024	16	2.319429	0.0514	0.8597	1.3132	2.595614
2048	512	0.25	50	1024	16	3.672272	0.0514	0.8594	1.3161	2.396296
2048	614	0.3	50	1024	16	4.352767	0.0504	0.8417	1.4973	2.473640
2048	849	0.4	50	1024	14	5.844768	0.0496	0.8283	1.6365	2.437463
2048	1024	0.5	50	1024	13	8.951393	0.0495	0.8274	1.6458	2.359380
2048	1229	0.6	50	1024	13	11.074836	0.0469	0.7845	2.1084	2.573343
2048	1434	0.7	50	1024	13	14.574213	0.0468	0.7824	2.1318	2.609980
2048	1536	0.75	50	1024	11	15.649662	0.0468	0.7816	2.1405	2.559536
2048	1638	0.8	50	1024	11	16.879158	0.0468	0.7816	2.1406	2.556170
2048	1843	0.9	50	1024	11	20.535287	0.0468	0.7816	2.1406	2.569219
2048	2048	1.0	50	1024	9	22.892901	0.0468	0.7815	2.1409	2.513244

**Table 9:** Performance analysis of the proposed db9 wavelet based sensing matrix

Length of signal (*N*)	Number of measurements (*m*)	Compression ratio (CR = *m/N*)	Sparsity level = (*k/N*) × 100 (%)	No. of non-zeros (*k*)	No. of iterations required	Signal reconstruction time (s)	RMSE	Relative error	SNR (db)	Construction time for sensing matrix (s)
2048	205	0.1	50	1024	15	1.252129	0.0583	0.9750	0.2203	2.649478
2048	410	0.2	50	1024	17	2.527130	0.0518	0.8660	1.2492	2.557930
2048	512	0.25	50	1024	17	3.972292	0.0518	0.8652	1.2582	2.531926
2048	614	0.3	50	1024	16	4.221050	0.0490	0.8182	1.7425	2.572460
2048	849	0.4	50	1024	15	6.270259	0.0478	0.7990	1.9495	2.683571
2048	1024	0.5	50	1024	14	9.593764	0.0478	0.7983	1.9571	2.533085
2048	1229	0.6	50	1024	13	11.075619	0.0470	0.7853	2.0992	2.499444
2048	1434	0.7	50	1024	13	14.518974	0.0468	0.7824	2.1309	2.539711
2048	1536	0.75	50	1024	12	17.178638	0.0468	0.7816	2.1405	2.489187
2048	1638	0.8	50	1024	12	18.043134	0.0468	0.7816	2.1406	2.506067
2048	1843	0.9	50	1024	12	22.370874	0.0468	0.7816	2.1407	2.569387
2048	2048	1.0	50	1024	9	22.751732	0.0468	0.7815	2.1409	2.497201

**Table 10:** Performance analysis of the proposed db10 wavelet based sensing matrix

Length of signal (*N*)	Number of measurements (*m*)	Compression ratio (CR = *m/N*)	Sparsity level = (*k/N*) × 100 (%)	No. of non-zeros (*k*)	No. of iterations required	Signal reconstruction time (s)	RMSE	Relative error	SNR (db)	Construction time for sensing matrix (s)
2048	205	0.1	50	1024	16	1.281382	0.0585	0.9774	0.1985	2.584058
2048	410	0.2	50	1024	16	2.331942	0.0521	0.8711	1.1984	2.523152
2048	512	0.25	50	1024	16	3.752369	0.0521	0.8707	1.2024	2.514315
2048	614	0.3	50	1024	16	4.242975	0.0488	0.8153	1.7740	2.570737
2048	849	0.4	50	1024	14	5.853003	0.0479	0.7997	1.9418	2.581421
2048	1024	0.5	50	1024	14	9.486588	0.0478	0.7993	1.9454	2.581856
2048	1229	0.6	50	1024	14	11.080416	0.0471	0.7877	2.0723	2.593704
2048	1434	0.7	50	1024	13	14.440796	0.0468	0.7824	2.1310	2.546346
2048	1536	0.75	50	1024	12	17.799127	0.0468	0.7816	2.1406	2.523620
2048	1638	0.8	50	1024	11	16.568279	0.0468	0.7816	2.1406	2.484885
2048	1843	0.9	50	1024	11	20.629636	0.0468	0.7816	2.1407	2.518902
2048	2048	1.0	50	1024	9	22.525032	0.0468	0.7815	2.1409	2.612855

**Fig. 2:**
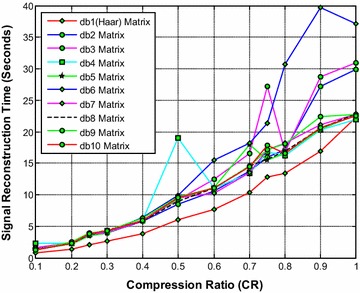
Effect of compression ratio on signal reconstruction time for different Daubechies wavelet sensing matrices

**Fig. 3:**
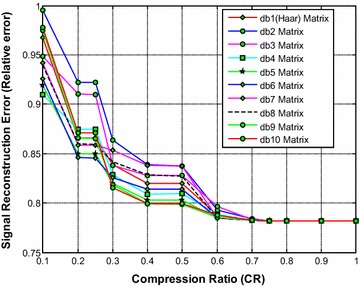
Effect of compression ratio on relative error for different Daubechies wavelet sensing matrices

**Fig. 4:**
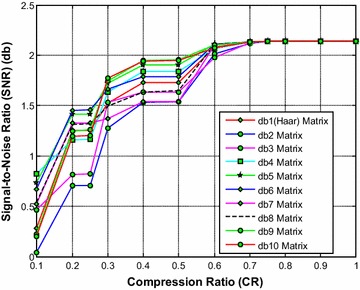
Effect of compression ratio on signal-to-noise ratio for different Daubechies wavelet sensing matrices

It is noted from Fig. 2 that the db1 (Haar) wavelet based sensing matrix requires less reconstruction time compared to all other Daubechies wavelet based sensing matrices. The second best choice will be db2 or db10, closely followed by the db9 wavelet based sensing matrix. From Fig. 3, it can be observed that the db10 wavelet based sensing matrix shows the minimum relative error compared to all other matrices. From Fig. 4, it can be observed that the db10 wavelet sensing matrix exhibits the high SNR (particularly from CR = 0.3 to CR = 1) compared to other sensing matrices.

Thus, it is evident from Figs. 2, 3 and 4 that overall the db10 wavelet based sensing matrix shows the good balance between signal reconstruction error and signal reconstruction time. Moreover, the db9 also shows a close performance to the db10 and may be the second best choice.

### Performance analysis of the Coiflets wavelet family based sensing matrices

This section demonstrates the performance analysis of the different DWT sensing matrices based on Coiflets wavelet family such as coif1, coif2, coif3, coif4 and coif5 (Tables 11, 12, 13, 14, 15).

**Table 11:** Performance analysis of the proposed coif1 wavelet based sensing matrix

Length of signal (*N*)	Number of measurements (*m*)	Compression ratio (CR = *m/N*)	Sparsity level = (*k/N*) × 100 (%)	No. of non-zeros (*k*)	No. of iterations required	Signal reconstruction time (s)	RMSE	Relative error	SNR (db)	Construction time for sensing matrix (s)
2048	205	0.1	50	1024	17	1.354238	0.0579	0.9668	0.2930	2.098881
2048	410	0.2	50	1024	15	2.874402	0.0550	0.9197	0.7271	2.041493
2048	512	0.25	50	1024	15	10.468670	0.0550	0.9196	0.7279	2.375475
2048	614	0.3	50	1024	14	5.955084	0.0519	0.8675	1.2346	2.308189
2048	849	0.4	50	1024	14	8.202050	0.0513	0.8579	1.3315	2.200196
2048	1024	0.5	50	1024	13	11.575710	0.0513	0.8568	1.3425	2.111921
2048	1229	0.6	50	1024	13	18.579869	0.0476	0.7952	1.9901	2.127905
2048	1434	0.7	50	1024	12	15.692999	0.0469	0.7830	2.1245	2.487333
2048	1536	0.75	50	1024	12	17.816849	0.0468	0.7817	2.1396	2.396088
2048	1638	0.8	50	1024	12	18.998337	0.0468	0.7816	2.1406	2.226200
2048	1843	0.9	50	1024	12	23.612591	0.0468	0.7816	2.1406	2.379104
2048	2048	1.0	50	1024	9	23.507435	0.0468	0.7815	2.1409	2.343294

**Table 12:** Performance analysis of the proposed coif2 wavelet based sensing matrix

Length of signal (*N*)	Number of measurements (*m*)	Compression ratio (CR = *m/N*)	Sparsity level = (*k/N*) × 100 (%)	No. of non-zeros (*k*)	No. of iterations required	Signal reconstruction time (s)	RMSE	Relative error	SNR (db)	Construction time for sensing matrix (s)
2048	205	0.1	50	1024	16	1.344408	0.0589	0.9847	0.1338	2.656092
2048	410	0.2	50	1024	15	2.311920	0.0542	0.9050	0.8674	2.634235
2048	512	0.25	50	1024	15	3.535317	0.0542	0.9052	0.8649	2.354271
2048	614	0.3	50	1024	15	4.017180	0.0531	0.8869	1.0428	2.595762
2048	849	0.4	50	1024	14	6.107924	0.0527	0.8812	1.0990	2.515127
2048	1024	0.5	50	1024	13	8.927963	0.0525	0.8773	1.1375	2.531853
2048	1229	0.6	50	1024	13	11.323331	0.0481	0.8035	1.9008	2.503729
2048	1434	0.7	50	1024	13	21.738856	0.0469	0.7832	2.1229	2.657620
2048	1536	0.75	50	1024	12	27.116146	0.0468	0.7817	2.1392	2.598041
2048	1638	0.8	50	1024	12	26.312156	0.0468	0.7816	2.1405	2.358481
2048	1843	0.9	50	1024	11	32.646767	0.0468	0.7816	2.1406	2.564254
2048	2048	1.0	50	1024	9	34.162602	0.0468	0.7815	2.1409	2.617750

**Table 13:** Performance analysis of the proposed coif3 wavelet based sensing matrix

Length of signal (*N*)	Number of measurements (*m*)	Compression ratio (CR = *m/N*)	Sparsity level = (*k/N*) × 100 (%)	No. of non-zeros (*k*)	No. of iterations required	Signal reconstruction time (s)	RMSE	Relative error	SNR (db)	Construction time for sensing matrix (s)
2048	205	0.1	50	1024	16	1.313659	0.0561	0.9375	0.5604	3.076415
2048	410	0.2	50	1024	16	2.300134	0.0529	0.8835	1.0761	2.883624
2048	512	0.25	50	1024	16	3.836235	0.0529	0.8834	1.0770	2.977264
2048	614	0.3	50	1024	15	4.095096	0.0492	0.8228	1.6944	2.991658
2048	849	0.4	50	1024	14	6.307728	0.0485	0.8105	1.8244	2.734366
2048	1024	0.5	50	1024	14	12.427927	0.0485	0.8105	1.8247	3.000826
2048	1229	0.6	50	1024	13	14.180720	0.0475	0.7931	2.0136	2.732994
2048	1434	0.7	50	1024	13	26.695054	0.0468	0.7824	2.1319	2.674142
2048	1536	0.75	50	1024	12	23.379583	0.0468	0.7816	2.1403	2.967887
2048	1638	0.8	50	1024	11	19.402939	0.0468	0.7816	2.1406	2.882567
2048	1843	0.9	50	1024	11	25.077965	0.0468	0.7816	2.1406	2.741203
2048	2048	1.0	50	1024	9	30.924540	0.0468	0.7815	2.1409	2.949021

**Table 14:** Performance analysis of the proposed coif4 wavelet based sensing matrix

Length of signal (*N*)	Number of measurements (*m*)	Compression ratio (CR = *m/N*)	Sparsity level = (*k/N*) × 100 (%)	No. of non-zeros (*k*)	No. of iterations required	Signal reconstruction time (s)	RMSE	Relative error	SNR (db)	Construction time for sensing matrix (s)
2048	205	0.1	50	1024	16	1.262131	0.0567	0.9480	0.4643	2.843727
2048	410	0.2	50	1024	17	2.439706	0.0524	0.8756	1.1538	2.830243
2048	512	0.25	50	1024	17	3.704519	0.0524	0.8757	1.1529	2.791644
2048	614	0.3	50	1024	16	4.097077	0.0491	0.8211	1.7123	2.743363
2048	849	0.4	50	1024	15	6.153716	0.0485	0.8105	1.8248	2.783660
2048	1024	0.5	50	1024	14	9.041550	0.0485	0.8103	1.8267	2.812775
2048	1229	0.6	50	1024	13	10.712781	0.0473	0.7911	2.0354	2.633953
2048	1434	0.7	50	1024	13	14.213775	0.0468	0.7824	2.1318	2.631650
2048	1536	0.75	50	1024	12	16.368278	0.0468	0.7816	2.1404	2.785540
2048	1638	0.8	50	1024	12	17.142833	0.0468	0.7816	2.1406	2.690370
2048	1843	0.9	50	1024	11	19.494505	0.0468	0.7816	2.1406	2.606268
2048	2048	1.0	50	1024	9	21.432247	0.0468	0.7815	2.1409	2.659983

**Table 15:** Performance analysis of the proposed coif5 wavelet based sensing matrix

Length of signal (*N*)	Number of measurements (*m*)	Compression ratio (CR = *m/N*)	Sparsity level = (*k/N*) × 100 (%)	No. of non-zeros (*k*)	No. of iterations required	Signal reconstruction time (s)	RMSE	Relative error	SNR (db)	Construction time for sensing matrix (s)
2048	205	0.1	50	1024	18	1.503436	0.0552	0.9229	0.6966	2.866005
2048	410	0.2	50	1024	17	2.491811	0.0509	0.8498	1.4132	2.895638
2048	512	0.25	50	1024	17	3.852817	0.0509	0.8500	1.4114	3.007931
2048	614	0.3	50	1024	16	4.188601	0.0489	0.8164	1.7616	3.009424
2048	849	0.4	50	1024	14	5.971268	0.0482	0.8060	1.8737	2.845210
2048	1024	0.5	50	1024	14	9.498384	0.0482	0.8058	1.8753	3.033461
2048	1229	0.6	50	1024	13	11.012061	0.0472	0.7889	2.0598	3.033341
2048	1434	0.7	50	1024	13	14.348884	0.0468	0.7824	2.1313	3.105129
2048	1536	0.75	50	1024	12	17.088544	0.0468	0.7816	2.1404	2.821626
2048	1638	0.8	50	1024	12	18.211057	0.0468	0.7816	2.1406	3.005033
2048	1843	0.9	50	1024	11	20.533120	0.0468	0.7816	2.1406	3.095259
2048	2048	1.0	50	1024	9	23.047740	0.0468	0.7815	2.1409	2.936779

**Fig. 5:**
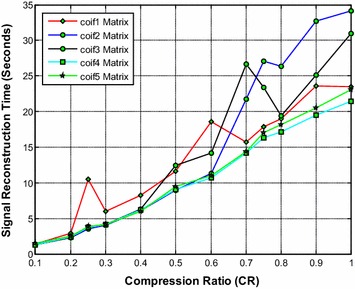
Effect of compression ratio on signal reconstruction time for different Coiflets wavelet sensing matrices

**Fig. 6:**
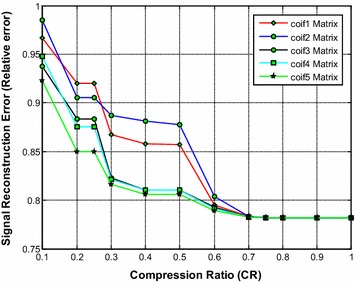
Effect of compression ratio on relative error for different Coiflets wavelet sensing matrices

**Fig. 7:**
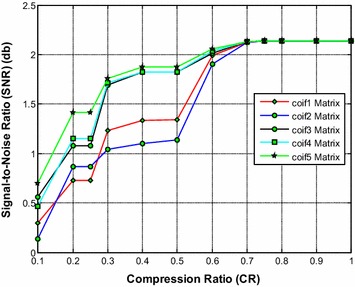
Effect of compression ratio on signal-to-noise ratio for different Coiflets wavelet sensing matrices

It is noted from Fig. 5 that the coif5 and coif4 wavelet based sensing matrix shows a close performance and requires the less reconstruction time compared to all other Coiflets wavelet based sensing matrices. From Fig. 6, it can be observed that coif5 wavelet based sensing matrix shows the minimum relative error compared to all other matrices. Also, from Fig. 7, it is seen that coif5 wavelet based sensing matrix exhibits the high SNR compared to other sensing matrices.

Thus, overall the coif5 wavelet based sensing matrix shows the good performance, since it requires the less reconstruction time, minimum relative error and the high SNR compared to other Coiflets wavelet based sensing matrices. In addition, the coif4 may be the second choice of sensing matrix.

### Performance analysis of the Symlets wavelet family based sensing matrices

This section demonstrates the performance analysis of the different DWT sensing matrices based on Symlets wavelet family such as sym4, sym5, sym6, sym7, sym8, sym9 and sym10 (Tables 16, 17, 18, 19, 20, 21, 22).

**Table 16:** Performance analysis of the proposed sym4 wavelet based sensing matrix

Length of signal (*N*)	Number of measurements (*m*)	Compression ratio (CR = *m/N*)	Sparsity level = (*k/N*) × 100 (%)	No. of non-zeros (*k*)	No. of iterations required	Signal reconstruction time (s)	RMSE	Relative error	SNR (db)	Construction time for sensing matrix (s)
2048	205	0.1	50	1024	15	1.426156	0.0606	1.0128	0.1101	2.219932
2048	410	0.2	50	1024	15	2.466473	0.0550	0.9186	0.7376	2.287469
2048	512	0.25	50	1024	15	4.034720	0.0550	0.9188	0.7360	2.408524
2048	614	0.3	50	1024	15	4.520797	0.0510	0.8516	1.3956	2.262042
2048	849	0.4	50	1024	14	6.883726	0.0503	0.8412	1.5016	2.461359
2048	1024	0.5	50	1024	14	9.275989	0.0503	0.8412	1.5015	2.600820
2048	1229	0.6	50	1024	13	10.699366	0.0479	0.8001	1.9374	2.083195
2048	1434	0.7	50	1024	13	13.702683	0.0468	0.7828	2.1269	2.117664
2048	1536	0.75	50	1024	12	16.142659	0.0468	0.7817	2.1393	2.303114
2048	1638	0.8	50	1024	12	17.024746	0.0468	0.7816	2.1405	2.259328
2048	1843	0.9	50	1024	12	21.118064	0.0468	0.7816	2.1406	2.369993
2048	2048	1.0	50	1024	9	21.227179	0.0468	0.7815	2.1409	2.248962

**Table 17:** Performance analysis of the proposed sym5 wavelet based sensing matrix

Length of signal (*N*)	Number of measurements (*m*)	Compression ratio (CR = *m/N*)	Sparsity level = (*k/N*) × 100 (%)	No. of non-zeros (*k*)	No. of iterations required	Signal reconstruction time (s)	RMSE	Relative error	SNR (db)	Construction time for sensing matrix (s)
2048	205	0.1	50	1024	18	1.562108	0.0587	0.9813	0.1644	2.474725
2048	410	0.2	50	1024	16	2.410647	0.0525	0.8767	1.1428	2.422995
2048	512	0.25	50	1024	15	3.363769	0.0524	0.8764	1.1460	2.414940
2048	614	0.3	50	1024	14	3.633501	0.0487	0.8139	1.7885	2.401602
2048	849	0.4	50	1024	14	5.901087	0.0485	0.8100	1.8307	2.285970
2048	1024	0.5	50	1024	13	8.629663	0.0484	0.8094	1.8370	2.270979
2048	1229	0.6	50	1024	12	10.436658	0.0476	0.7951	1.9910	2.295386
2048	1434	0.7	50	1024	12	12.997505	0.0468	0.7825	2.1299	2.416593
2048	1536	0.75	50	1024	12	16.543217	0.0468	0.7816	2.1400	2.226996
2048	1638	0.8	50	1024	11	15.839222	0.0468	0.7816	2.1407	2.255090
2048	1843	0.9	50	1024	11	19.887183	0.0468	0.7816	2.1407	2.431380
2048	2048	1.0	50	1024	9	21.319450	0.0468	0.7815	2.1409	2.266367

**Table 18:** Performance analysis of the proposed sym6 wavelet based sensing matrix

Length of signal (*N*)	Number of measurements (*m*)	Compression ratio (CR = *m/N*)	Sparsity level = (*k/N*) × 100 (%)	No. of non-zeros (*k*)	No. of iterations required	Signal reconstruction time (s)	RMSE	Relative error	SNR (db)	Construction time for sensing matrix (s)
2048	205	0.1	50	1024	16	1.337284	0.0568	0.9486	0.4587	2.278876
2048	410	0.2	50	1024	16	2.354315	0.0519	0.8665	1.2450	2.291421
2048	512	0.25	50	1024	15	3.403486	0.0520	0.8696	1.2132	2.495518
2048	614	0.3	50	1024	15	3.900882	0.0506	0.8450	1.4632	2.513799
2048	849	0.4	50	1024	14	5.894927	0.0495	0.8274	1.6455	2.444608
2048	1024	0.5	50	1024	14	9.191334	0.0495	0.8274	1.6452	2.282627
2048	1229	0.6	50	1024	13	10.874663	0.0470	0.7858	2.0939	2.354739
2048	1434	0.7	50	1024	13	13.986553	0.0468	0.7825	2.1300	2.269408
2048	1536	0.75	50	1024	12	16.289155	0.0468	0.7816	2.1404	2.324362
2048	1638	0.8	50	1024	12	17.002074	0.0468	0.7816	2.1406	2.325569
2048	1843	0.9	50	1024	12	21.603990	0.0468	0.7816	2.1407	2.409376
2048	2048	1.0	50	1024	9	21.488590	0.0468	0.7815	2.1409	2.344651

**Table 19:** Performance analysis of the proposed sym7 wavelet based sensing matrix

Length of signal (*N*)	Number of measurements (*m*)	Compression ratio (CR = *m/N*)	Sparsity level = (*k/N*) × 100 (%)	No. of non-zeros (*k*)	No. of iterations required	Signal reconstruction time (s)	RMSE	Relative error	SNR (db)	Construction time for sensing matrix (s)
2048	205	0.1	50	1024	19	1.604935	0.0549	0.9178	0.7450	2.385164
2048	410	0.2	50	1024	16	2.270355	0.0530	0.8860	1.0514	2.368900
2048	512	0.25	50	1024	16	3.611425	0.0530	0.8857	1.0539	2.390055
2048	614	0.3	50	1024	14	3.719657	0.0494	0.8259	1.6620	2.400246
2048	849	0.4	50	1024	14	5.943942	0.0486	0.8126	1.8025	2.379669
2048	1024	0.5	50	1024	14	9.231755	0.0486	0.8125	1.8037	2.626292
2048	1229	0.6	50	1024	14	11.697056	0.0477	0.7968	1.9732	2.395632
2048	1434	0.7	50	1024	13	14.038048	0.0468	0.7824	2.1319	2.547235
2048	1536	0.75	50	1024	12	16.379478	0.0468	0.7816	2.1398	2.541034
2048	1638	0.8	50	1024	12	17.139381	0.0468	0.7816	2.1406	2.299743
2048	1843	0.9	50	1024	11	19.813613	0.0468	0.7816	2.1406	2.628218
2048	2048	1.0	50	1024	9	21.393741	0.0468	0.7815	2.1409	2.435649

**Table 20:** Performance analysis of the proposed sym8 wavelet based sensing matrix

Length of signal (*N*)	Number of measurements (*m*)	Compression ratio (CR = *m/N*)	Sparsity level = (*k/N*) × 100 (%)	No. of non-zeros (*k*)	No. of iterations required	Signal reconstruction time (s)	RMSE	Relative error	SNR (db)	Construction time for sensing matrix (s)
2048	205	0.1	50	1024	16	1.337804	0.0591	0.9881	0.1037	2.398959
2048	410	0.2	50	1024	16	2.289342	0.0525	0.8775	1.1354	2.449329
2048	512	0.25	50	1024	16	3.605457	0.0525	0.8774	1.1361	2.413914
2048	614	0.3	50	1024	15	3.899311	0.0490	0.8194	1.7296	2.367131
2048	849	0.4	50	1024	14	5.839426	0.0489	0.8165	1.7613	2.414094
2048	1024	0.5	50	1024	14	9.219034	0.0489	0.8167	1.7590	2.477653
2048	1229	0.6	50	1024	13	10.916775	0.0476	0.7953	1.9899	2.412281
2048	1434	0.7	50	1024	13	14.105790	0.0468	0.7823	2.1324	2.411482
2048	1536	0.75	50	1024	12	16.337553	0.0468	0.7816	2.1403	2.425477
2048	1638	0.8	50	1024	11	15.876267	0.0468	0.7816	2.1406	2.452373
2048	1843	0.9	50	1024	11	19.838644	0.0468	0.7816	2.1406	2.439456
2048	2048	1.0	50	1024	9	21.412296	0.0468	0.7815	2.1409	2.481033

**Table 21:** Performance analysis of the proposed sym9 wavelet based sensing matrix

Length of signal (*N*)	Number of measurements (*m*)	Compression ratio (CR = *m/N*)	Sparsity level = (*k/N*) × 100 (%)	No. of non-zeros (*k*)	No. of iterations required	Signal reconstruction time (s)	RMSE	Relative error	SNR (db)	Construction time for sensing matrix (s)
2048	205	0.1	50	1024	17	1.433657	0.0582	0.9731	0.2364	2.566095
2048	410	0.2	50	1024	16	2.362390	0.0520	0.8695	1.2149	2.566405
2048	512	0.25	50	1024	16	3.703235	0.0520	0.8690	1.2191	2.529453
2048	614	0.3	50	1024	15	3.947898	0.0493	0.8243	1.6788	2.580208
2048	849	0.4	50	1024	14	5.824850	0.0481	0.8038	1.8975	2.566482
2048	1024	0.5	50	1024	13	8.527638	0.0481	0.8038	1.8973	2.627600
2048	1229	0.6	50	1024	12	9.961474	0.0470	0.7850	2.1023	2.621438
2048	1434	0.7	50	1024	12	12.963035	0.0468	0.7822	2.1333	2.582570
2048	1536	0.75	50	1024	12	16.468013	0.0468	0.7816	2.1406	2.628998
2048	1638	0.8	50	1024	11	15.820224	0.0468	0.7816	2.1406	2.705263
2048	1843	0.9	50	1024	11	19.915216	0.0468	0.7816	2.1407	2.695629
2048	2048	1.0	50	1024	9	21.477969	0.0468	0.7815	2.1409	2.618640

**Table 22:** Performance analysis of the proposed sym10 wavelet based sensing matrix

Length of signal (*N*)	Number of measurements (*m*)	Compression ratio (CR = *m/N*)	Sparsity level = (*k/N*) × 100 (%)	No. of non-zeros (*k*)	No. of iterations required	Signal reconstruction time (s)	RMSE	Relative error	SNR (db)	Construction time for sensing matrix (s)
2048	205	0.1	50	1024	17	1.479121	0.0560	0.9351	0.5831	2.708358
2048	410	0.2	50	1024	16	2.348326	0.0513	0.8578	1.3323	2.607475
2048	512	0.25	50	1024	16	3.762705	0.0513	0.8580	1.3298	2.729382
2048	614	0.3	50	1024	16	4.328904	0.0490	0.8192	1.7321	2.819432
2048	849	0.4	50	1024	14	6.057941	0.0479	0.8003	1.9353	2.719236
2048	1024	0.5	50	1024	14	9.516700	0.0479	0.8010	1.9273	2.715742
2048	1229	0.6	50	1024	13	11.268634	0.0470	0.7853	2.0992	2.484238
2048	1434	0.7	50	1024	12	13.607226	0.0468	0.7822	2.1333	2.680826
2048	1536	0.75	50	1024	12	17.225086	0.0468	0.7816	2.1405	2.634090
2048	1638	0.8	50	1024	12	18.192019	0.0468	0.7816	2.1406	2.628795
2048	1843	0.9	50	1024	12	22.716723	0.0468	0.7816	2.1406	2.709115
2048	2048	1.0	50	1024	9	22.909145	0.0468	0.7815	2.1409	2.612016

**Fig. 8:**
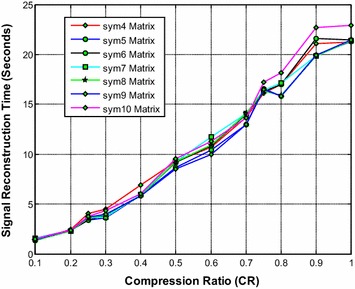
Effect of compression ratio on signal reconstruction time for different Symlets wavelet sensing matrices

**Fig. 9:**
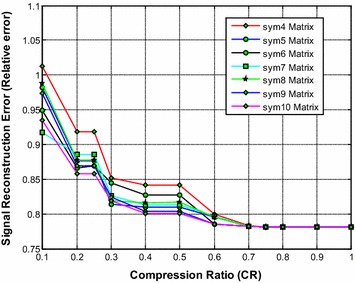
Effect of compression ratio on relative error for different Symlets wavelet sensing matrices

**Fig. 10:**
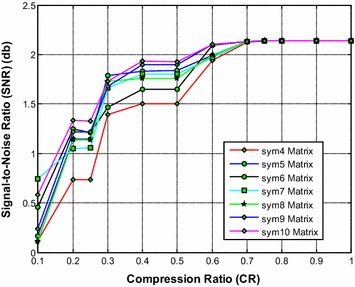
Effect of compression ratio on signal-to-noise ratio for different Symlets wavelet sensing matrices

It is noted from Fig. 8 that the sym9 wavelet based sensing matrix requires the less reconstruction time compared to all other Symlets wavelet based sensing matrices. Furthermore, the sym5 also shows a very close performance to that of the sym9 wavelet based sensing matrix. From Fig. 9, it can be observed that the sym9 and the sym10 wavelet based sensing matrices almost demonstrate similar performance with minimum relative error compared to all other matrices. Also, from Fig. 10, it is observed that the sym9 and the sym10 wavelet based sensing matrices nearly shows similar performance and exhibits the high SNR compared to other sensing matrices.

Thus, it is evident from Figs. 9 and 10 that overall the sym9 wavelet sensing matrix demonstrates the less reconstruction time and the less relative error, and thus exhibits the good performance compared to other Symlets wavelet based sensing matrices. Moreover, the sym10 may be the second choice of sensing matrix followed by the sym5.

### Performance analysis of the Beylkin, Vaidyanathan and Battle wavelet family based sensing matrices

This section shows the performance analysis of the different DWT sensing matrices based on Beylkin, Vaidyanathan, and Battle1, Battle3 and Battle5 wavelet families (Tables 23, 24, 25, 26, 27).

**Table 23:** Performance analysis of the proposed Beylkin wavelet based sensing matrix

Length of signal (*N*)	Number of measurements (*m*)	Compression ratio (CR = *m/N*)	Sparsity level = (*k/N*) × 100 (%)	No. of non-zeros (*k*)	No. of iterations required	Signal reconstruction time (s)	RMSE	Relative error	SNR (db)	Construction time for sensing matrix (s)
2048	205	0.1	50	1024	17	1.351917	0.0551	0.9205	0.7196	2.635292
2048	410	0.2	50	1024	16	2.313493	0.0522	0.8726	1.1837	2.500319
2048	512	0.25	50	1024	16	3.655763	0.0522	0.8722	1.1878	2.582726
2048	614	0.3	50	1024	16	4.267978	0.0490	0.8193	1.7316	2.668384
2048	849	0.4	50	1024	14	5.865898	0.0483	0.8064	1.8686	2.501577
2048	1024	0.5	50	1024	14	9.541513	0.0482	0.8056	1.8774	2.626348
2048	1229	0.6	50	1024	13	11.113448	0.0469	0.7838	2.1163	2.464044
2048	1434	0.7	50	1024	13	14.567693	0.0468	0.7822	2.1341	2.504371
2048	1536	0.75	50	1024	12	17.19308	0.0468	0.7816	2.1405	2.527696
2048	1638	0.8	50	1024	12	18.081755	0.0468	0.7816	2.1406	2.516479
2048	1843	0.9	50	1024	12	22.526654	0.0468	0.7816	2.1407	2.523531
2048	2048	1.0	50	1024	9	22.950703	0.0468	0.7815	2.1409	2.466272

**Table 24:** Performance analysis of the proposed Vaidyanathan wavelet based sensing matrix

Length of signal (*N*)	Number of measurements (*m*)	Compression ratio (CR = *m/N*)	Sparsity level = (*k/N*) × 100 (%)	No. of non-zeros (*k*)	No. of iterations required	Signal reconstruction time (s)	RMSE	Relative error	SNR (db)	Construction time for sensing matrix (s)
2048	205	0.1	50	1024	19	1.707644	0.0702	1.1724	1.3812	2.912996
2048	410	0.2	50	1024	19	3.459463	0.0580	0.9684	0.2785	3.332869
2048	512	0.25	50	1024	19	5.666164	0.0580	0.9685	0.2782	3.036002
2048	614	0.3	50	1024	18	5.760958	0.0513	0.8578	1.3320	3.034769
2048	849	0.4	50	1024	15	7.316646	0.0485	0.8112	1.8177	2.969049
2048	1024	0.5	50	1024	14	10.855239	0.0486	0.8123	1.8053	3.075962
2048	1229	0.6	50	1024	13	13.417422	0.0474	0.7924	2.0214	3.441779
2048	1434	0.7	50	1024	13	19.093624	0.0468	0.7822	2.1339	3.483836
2048	1536	0.75	50	1024	12	22.608453	0.0468	0.7816	2.1405	3.022221
2048	1638	0.8	50	1024	12	34.970415	0.0468	0.7816	2.1406	3.586396
2048	1843	0.9	50	1024	12	49.314450	0.0468	0.7816	2.1407	3.515476
2048	2048	1.0	50	1024	9	34.943702	0.0468	0.7815	2.1409	3.060519

**Table 25:** Performance analysis of the proposed Battle1 wavelet based sensing matrix

Length of signal (*N*)	Number of measurements (*m*)	Compression ratio (CR = *m/N*)	Sparsity level = (*k/N*) × 100 (%)	No. of non-zeros (*k*)	No. of iterations required	Signal reconstruction time (s)	RMSE	Relative error	SNR (db)	Construction time for sensing matrix (s)
2048	205	0.1	50	1024	15	1.453478	0.0633	1.0577	0.4873	2.794529
2048	410	0.2	50	1024	14	2.113667	0.0556	0.9283	0.6459	2.953615
2048	512	0.25	50	1024	15	3.530676	0.0560	0.9363	0.5713	2.769843
2048	614	0.3	50	1024	14	3.957949	0.0514	0.8598	1.3125	2.621663
2048	849	0.4	50	1024	14	7.280928	0.0513	0.8571	1.3399	2.846945
2048	1024	0.5	50	1024	14	9.777801	0.0514	0.8587	1.3230	2.858507
2048	1229	0.6	50	1024	13	11.850497	0.0480	0.8013	1.9241	2.643100
2048	1434	0.7	50	1024	13	15.195628	0.0468	0.7827	2.1278	2.621185
2048	1536	0.75	50	1024	12	17.503087	0.0468	0.7817	2.1387	2.804361
2048	1638	0.8	50	1024	11	17.421545	0.0468	0.7816	2.1406	2.835200
2048	1843	0.9	50	1024	11	28.179929	0.0468	0.7816	2.1406	2.869269
2048	2048	1.0	50	1024	9	32.640965	0.0468	0.7815	2.1409	3.447333

**Table 26:** Performance analysis of the proposed Battle3 wavelet based sensing matrix

Length of signal (*N*)	Number of measurements (*m*)	Compression ratio (CR = *m/N*)	Sparsity level = (*k/N*) × 100 (%)	No. of non-zeros (*k*)	No. of iterations required	Signal reconstruction time (s)	RMSE	Relative error	SNR (db)	Construction time for sensing matrix (s)
2048	205	0.1	50	1024	21	2.229166	0.0776	1.2960	2.2520	4.184386
2048	410	0.2	50	1024	17	3.000747	0.0619	1.0350	0.2991	4.037021
2048	512	0.25	50	1024	17	6.762783	0.0619	1.0350	0.2984	4.214151
2048	614	0.3	50	1024	17	5.495381	0.0576	0.9626	0.3313	3.779477
2048	849	0.4	50	1024	15	7.920600	0.0538	0.8989	0.9261	3.706719
2048	1024	0.5	50	1024	14	11.331212	0.0538	0.8995	0.9199	3.945231
2048	1229	0.6	50	1024	13	13.909190	0.0485	0.8106	1.8235	3.764331
2048	1434	0.7	50	1024	12	19.831917	0.0468	0.7823	2.1321	3.777555
2048	1536	0.75	50	1024	12	34.010388	0.0468	0.7816	2.1404	4.464107
2048	1638	0.8	50	1024	12	30.235744	0.0468	0.7816	2.1406	3.855705
2048	1843	0.9	50	1024	11	40.190816	0.0468	0.7816	2.1406	4.003610
2048	2048	1.0	50	1024	9	45.789195	0.0468	0.7815	2.1409	3.726738

**Table 27:** Performance analysis of the proposed Battle5 wavelet based sensing matrix

Length of signal (*N*)	Number of measurements (*m*)	Compression ratio (CR = *m/N*)	Sparsity level = (*k/N*) × 100 (%)	No. of non-zeros (*k*)	No. of iterations required	Signal reconstruction time (s)	RMSE	Relative error	SNR (db)	Construction time for sensing matrix (s)
2048	205	0.1	50	1024	16	1.547147	0.0587	0.9809	0.1672	4.355811
2048	410	0.2	50	1024	16	2.776326	0.0517	0.8633	1.2764	4.523638
2048	512	0.25	50	1024	17	4.876061	0.0518	0.8648	1.2619	4.258686
2048	614	0.3	50	1024	16	5.068130	0.0492	0.8226	1.6961	4.216043
2048	849	0.4	50	1024	15	8.173479	0.0479	0.7999	1.9398	4.653944
2048	1024	0.5	50	1024	13	22.056223	0.0480	0.8013	1.9240	4.196677
2048	1229	0.6	50	1024	13	13.576984	0.0470	0.7858	2.0936	4.174972
2048	1434	0.7	50	1024	13	18.405100	0.0468	0.7821	2.1350	4.355072
2048	1536	0.75	50	1024	12	22.430252	0.0468	0.7816	2.1404	4.268081
2048	1638	0.8	50	1024	11	28.325501	0.0468	0.7816	2.1406	4.269212
2048	1843	0.9	50	1024	11	38.805249	0.0468	0.7816	2.1407	4.623429
2048	2048	1.0	50	1024	9	41.186350	0.0468	0.7815	2.1409	4.283169

**Fig. 11:**
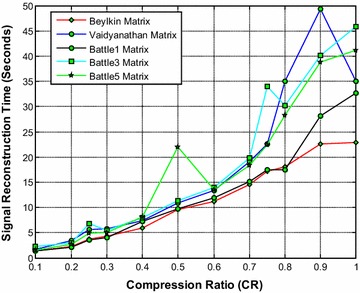
Effect of compression ratio on signal reconstruction time for Beylkin, Vaidyanathan and Battle wavelet sensing matrices

**Fig. 12:**
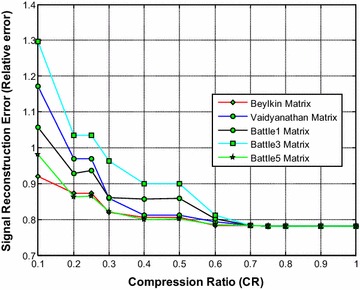
Effect of compression ratio on relative error for Beylkin, Vaidyanathan and Battle wavelet sensing matrices

**Fig. 13:**
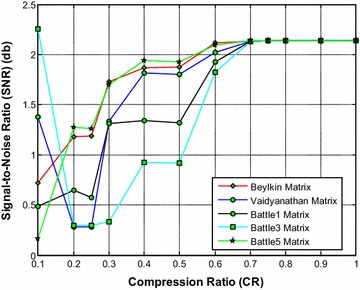
Effect of compression ratio on signal-to-noise ratio for Beylkin, Vaidyanathan and Battle wavelet sensing matrices

Figure 11 shows that the Beylkin wavelet based sensing matrix requires the less reconstruction time compared to all other Symlets wavelet based sensing matrices. From Fig. 12, it can be observed that the Beylkin and the Battle5 wavelet based sensing matrices shows a very close performance with minimum relative error compared to all other matrices. Also, from Fig. 13, it can be seen that the Beylkin and the Battle5 wavelet based sensing matrices shows a very comparable performance and exhibits the high SNR compared to other sensing matrices.

Thus, it can be noted from Figs. 11, 12 and 13 that overall the Beylkin wavelet sensing matrix demonstrates the less reconstruction time and relative error, and thus exhibits the good performance compared to other wavelet based sensing matrices. However, the Battle5 shows a close performance and may be the second best choice of sensing matrix.

### Performance analysis of the best-proposed DWT based sensing matrices namely: Beylkin, db10, coif5 and sym9 wavelet family

This section illustrates the performance analysis of the best-proposed DWT sensing matrices namely: Beylkin, db10, coif5 and sym9 wavelet families.

**Fig. 14:**
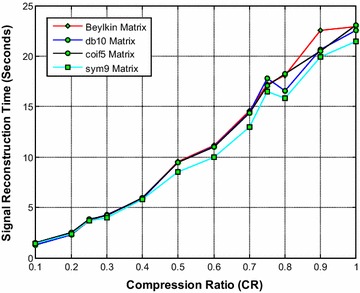
Effect of compression ratio on signal reconstruction time for the best DWT based sensing matrices

**Fig. 15:**
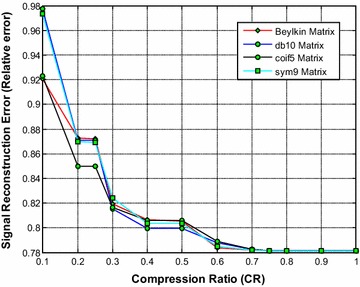
Effect of compression ratio on relative error for the best DWT based sensing matrices

**Fig. 16:**
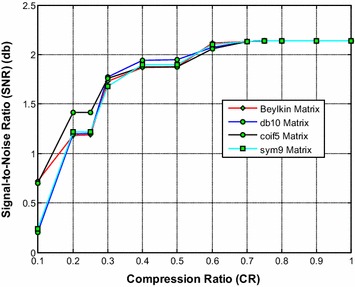
Effect of compression ratio on signal-to-noise ratio for the best DWT based sensing matrices

Figure 14 shows that the sym9 wavelet based sensing matrix clearly outperforms the Beylkin, db10, and the coif5 wavelet based sensing matrices in terms of signal reconstruction time. From Fig. 15, it can be observed that the db10 shows the good performance over CR = 0.3–0.5; however overall the sym9 wavelet based sensing matrices shows the good (from CR = 0.5–1.0) and comparable performance with db10. Also, from Fig. 16, it can be observed that the db10 and sym9 wavelet based sensing matrices shows a comparable performance and exhibits the high SNR compared to other sensing matrices. In addition, the sym9 wavelet based sensing matrix shows an edge over db10 from the CR = 0.5–1.0.

Thus, it can be evident from Figs. 14, 15 and 16 that overall the sym9 wavelet based sensing matrix shows the superior performance compared to the Beylkin, db10 and the coif5 wavelet based sensing matrices in views of signal reconstruction time and relative error. Furthermore, the db10 may be the second best choice of sensing matrix.

### Performance analysis of the best-proposed sym9 wavelet based sensing matrix with state-of-the-art random and deterministic sensing matrices

This section illustrates the comparative analysis of the proposed sym9 wavelet based sensing matrix and state-of-the-art random sensing matrices such as Gaussian, Uniform, Toeplitz, Circulant and Hadamard matrix along with deterministic sensing matrices such as the DCT and the sparse binary sensing matrices for speech signal compression (Tables 28, 29, 30, 31, 32, 33, 34).

**Table 28:** Performance analysis of the random Gaussian sensing matrix

Length of signal (*N*)	Number of measurements (*m*)	Compression ratio (CR = *m/N*)	Sparsity level = (*k/N*) × 100 (%)	No. of non-zeros (*k*)	No. of iterations required	Signal reconstruction time (s)	RMSE	Relative error	SNR (db)	Construction time for sensing matrix (s)
2048	205	0.1	50	1024	21	1.614613	0.0529	0.8846	0.6667	1.096733
2048	410	0.2	50	1024	22	3.101767	0.0506	0.8459	1.4526	0.748022
2048	512	0.25	50	1024	21	4.639830	0.0488	0.8163	1.6802	1.942504
2048	614	0.3	50	1024	20	5.067599	0.0488	0.8149	1.8149	2.142537
2048	849	0.4	50	1024	21	8.240858	0.0476	0.7949	2.0516	4.909329
2048	1024	0.5	50	1024	20	12.792057	0.0470	0.7847	2.1202	11.635789
2048	1229	0.6	50	1024	21	17.375626	0.0468	0.7817	2.1431	14.081669
2048	1434	0.7	50	1024	22	23.472323	0.0468	0.7816	2.1388	34.631693
2048	1536	0.75	50	1024	24	33.471279	0.0468	0.7816	2.1416	39.194676
2048	1638	0.8	50	1024	27	38.907176	0.0468	0.7816	2.1408	43.476185
2048	1843	0.9	50	1024	23	41.156261	0.0468	0.7816	2.1409	51.096753
2048	2048	1.0	50	1024	9	22.129988	0.0468	0.7815	2.1409	57.755398

**Table 29:** Performance analysis of the random Uniform sensing matrix

Length of signal (*N*)	Number of measurements (*m*)	Compression ratio (CR = *m/N*)	Sparsity level = (*k/N*) × 100 (%)	No. of non-zeros (*k*)	No. of iterations required	Signal reconstruction time (s)	RMSE	Relative error	SNR (db)	Construction time for sensing matrix (s)
2048	205	0.1	50	1024	28	2.235539	0.0634	1.0600	1.0296	0.223625
2048	410	0.2	50	1024	25	3.600845	0.0537	0.8966	0.8152	0.738591
2048	512	0.25	50	1024	25	5.745004	0.0516	0.8616	0.8698	1.917257
2048	614	0.3	50	1024	25	6.434319	0.0496	0.8281	1.6951	2.113897
2048	849	0.4	50	1024	23	9.443467	0.0474	0.7924	1.9810	5.332054
2048	1024	0.5	50	1024	23	15.986578	0.0470	0.7857	2.0911	11.975395
2048	1229	0.6	50	1024	24	19.727464	0.0468	0.7823	2.1297	14.165440
2048	1434	0.7	50	1024	24	25.920229	0.0468	0.7816	2.1401	34.968949
2048	1536	0.75	50	1024	23	72.481383	0.0468	0.7816	2.1407	48.408631
2048	1638	0.8	50	1024	20	29.193737	0.0468	0.7816	2.1407	93.407060
2048	1843	0.9	50	1024	17	32.821700	0.0468	0.7816	2.1408	51.437677
2048	2048	1.0	50	1024	9	22.395440	0.0468	0.7815	2.1409	57.803101

**Table 30:** Performance analysis of the random Hadamard sensing matrix

Length of signal (*N*)	Number of measurements (*m*)	Compression ratio (CR = *m/N*)	Sparsity level = (*k/N*) × 100 (%)	No. of non-zeros (*k*)	No. of iterations required	Signal reconstruction time (s)	RMSE	Relative error	SNR (db)	Construction time for sensing matrix (s)
2048	205	0.1	50	1024	24	2.160564	0.0543	0.9068	0.6816	0.175290
2048	410	0.2	50	1024	21	3.352213	0.0508	0.8486	1.5002	0.195649
2048	512	0.25	50	1024	21	5.245114	0.0488	0.8162	1.7910	0.227224
2048	614	0.3	50	1024	14	4.059570	0.0476	0.7959	1.7840	0.203726
2048	849	0.4	50	1024	14	7.103978	0.0483	0.8075	2.1509	0.238574
2048	1024	0.5	50	1024	17	12.420312	0.0469	0.7838	2.1742	0.312728
2048	1229	0.6	50	1024	20	18.202941	0.0468	0.7814	2.1341	0.262874
2048	1434	0.7	50	1024	24	28.330063	0.0468	0.7816	2.1400	0.281225
2048	1536	0.75	50	1024	21	32.017893	0.0467	0.7805	2.1406	0.400085
2048	1638	0.8	50	1024	23	47.091449	0.0467	0.7803	2.1407	0.304155
2048	1843	0.9	50	1024	26	70.684798	0.0467	0.7805	2.1408	0.318110
2048	2048	1.0	50	1024	9	31.953103	0.0468	0.7815	2.1408	0.463961

**Table 31:** Performance analysis of the random Toeplitz sensing matrix

Length of signal (*N*)	Number of measurements (*m*)	Compression ratio (CR = *m/N*)	Sparsity level = (*k/N*) × 100 (%)	No. of non-zeros (*k*)	No. of iterations required	Signal reconstruction time (s)	RMSE	Relative error	SNR (db)	Construction time for sensing matrix (s)
2048	205	0.1	50	1024	21	1.756908	0.0536	0.8957	0.9104	0.409620
2048	410	0.2	50	1024	22	3.233689	0.0498	0.8315	1.5469	0.429934
2048	512	0.25	50	1024	21	4.788428	0.0493	0.8232	1.7366	0.469536
2048	614	0.3	50	1024	20	5.355458	0.0483	0.8068	1.8057	0.450123
2048	849	0.4	50	1024	20	8.429285	0.0474	0.7918	2.0918	0.471607
2048	1024	0.5	50	1024	20	13.439433	0.0469	0.7841	2.1270	0.544915
2048	1229	0.6	50	1024	21	17.961354	0.0467	0.7797	2.1501	0.503870
2048	1434	0.7	50	1024	21	23.162537	0.0467	0.7806	2.1523	0.524939
2048	1536	0.75	50	1024	23	32.312776	0.0467	0.7806	2.1511	0.626275
2048	1638	0.8	50	1024	24	35.325303	0.0467	0.7807	2.1490	0.549642
2048	1843	0.9	50	1024	27	49.591100	0.0467	0.7812	2.1453	0.562993
2048	2048	1.0	50	1024	6	18.351954	0.0468	0.7815	2.1409	0.695494

**Table 32:** Performance analysis of the random Circulant sensing matrix

Length of signal (*N*)	Number of measurements (*m*)	Compression ratio (CR = *m/N*)	Sparsity level = (*k/N*) × 100 (%)	No. of non-zeros (*k*)	No. of iterations required	Signal reconstruction time (s)	RMSE	Relative error	SNR (db)	Construction time for sensing matrix (s)
2048	205	0.1	50	1024	26	2.507042	0.0597	0.9968	0.4222	2.757110
2048	410	0.2	50	1024	24	3.729785	0.0510	0.8516	1.5673	1.755148
2048	512	0.25	50	1024	22	5.278771	0.0491	0.8206	1.7278	1.819234
2048	614	0.3	50	1024	19	5.237320	0.0480	0.8021	1.9582	1.813663
2048	849	0.4	50	1024	18	8.220705	0.0470	0.7846	2.1030	1.845629
2048	1024	0.5	50	1024	17	11.916562	0.0468	0.7825	2.1335	1.917508
2048	1229	0.6	50	1024	16	14.656704	0.0468	0.7816	2.1404	1.857957
2048	1434	0.7	50	1024	15	17.577009	0.0468	0.7816	2.1408	1.852721
2048	1536	0.75	50	1024	16	23.864195	0.0468	0.7816	2.1408	2.020327
2048	1638	0.8	50	1024	15	23.451971	0.0468	0.7816	2.1409	1.893177
2048	1843	0.9	50	1024	12	25.954229	0.0468	0.7815	2.1409	1.914215
2048	2048	1.0	50	1024	8	24.190480	0.0468	0.7815	2.1409	2.065995

**Table 33:** Performance analysis of the Deterministic DCT sensing matrix

Length of signal (*N*)	Number of measurements (*m*)	Compression ratio (CR = *m/N*)	Sparsity level = (*k/N*) × 100 (%)	No. of non-zeros (*k*)	No. of iterations required	Signal reconstruction time (s)	RMSE	Relative error	SNR (db)	Construction time for sensing matrix (s)
2048	205	0.1	50	1024	15	1.336874	0.0570	0.9532	0.4165	0.032836
2048	410	0.2	50	1024	16	2.309603	0.0537	0.8974	0.9405	0.060471
2048	512	0.25	50	1024	17	3.773514	0.0511	0.8543	1.3682	0.110442
2048	614	0.3	50	1024	16	4.122633	0.0510	0.8525	1.3858	0.087157
2048	849	0.4	50	1024	14	5.822766	0.0494	0.8259	1.6617	0.117717
2048	1024	0.5	50	1024	14	9.058112	0.0478	0.7981	1.9592	0.213083
2048	1229	0.6	50	1024	15	12.369809	0.0472	0.7889	2.0593	0.170241
2048	1434	0.7	50	1024	13	14.324836	0.0469	0.7832	2.1224	0.198500
2048	1536	0.75	50	1024	13	18.236878	0.0468	0.7825	2.1303	0.323367
2048	1638	0.8	50	1024	13	18.600745	0.0468	0.7820	2.1363	0.225549
2048	1843	0.9	50	1024	12	21.461995	0.0468	0.7816	2.1409	0.253978
2048	2048	1.00	50	1024	9	21.893476	0.0468	0.7815	2.1409	0.438310

**Table 34:** Performance analysis of the Deterministic Sparse Binary sensing matrix

Length of signal (*N*)	Number of measurements (*m*)	Compression ratio (CR = *m/N*)	Sparsity level = (*k/N*) × 100 (%)	No. of non-zeros (*k*)	No. of iterations required	Signal reconstruction time (s)	RMSE	Relative error	SNR (db)	Construction time for sensing matrix (s)
2048	205	0.1	50	1024	12	0.9278	0.0548	0.9154	0.7678	66.9190
2048	410	0.2	50	1024	13	1.8871	0.0503	0.8405	1.5090	207.1541
2048	512	0.25	50	1024	14	3.1431	0.0497	0.8308	1.6102	380.2697
2048	614	0.3	50	1024	15	4.8355	0.0487	0.8146	1.7809	502.1809
2048	849	0.4	50	1024	19	8.3895	0.0473	0.7906	2.0411	878.8051
2048	1024	0.5	50	1024	20	18.3753	0.0469	0.7843	2.1103	1271.5155
2048	1229	0.6	50	1024	23	25.4419	0.0468	0.7824	2.1313	1869.4049
2048	1434	0.7	50	1024	30	42.2472	0.0468	0.7816	2.1407	2913.4315
2048	1536	0.75	50	1024	28	57.3386	0.0468	0.7816	2.1408	2984.3214
2048	1638	0.8	50	1024	24	52.8342	0.0468	0.7816	2.1409	3575.2037
2048	1843	0.9	50	1024	17	50.7545	0.0468	0.7816	2.1409	4030.6333
2048	2048	1.0	50	1024	6	26.8845	0.0468	0.7815	2.1409	5856.4331

**Fig. 17:**
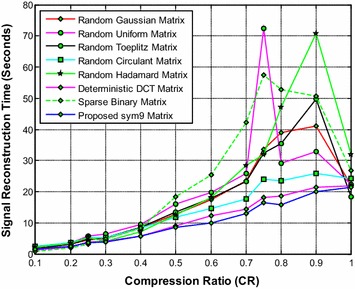
Effect of compression ratio on signal reconstruction time for the proposed sym9 matrix and state-of-the-art random and deterministic sensing matrices

**Fig. 18:**
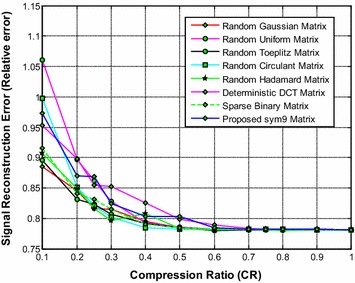
Effect of compression ratio on relative error for the proposed sym9 matrix and state-of-the-art random and deterministic sensing matrices

**Fig. 19:**
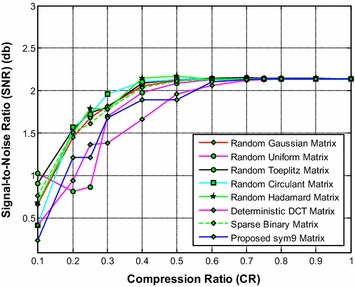
Effect of compression ratio on signal-to-noise ratio for the proposed sym9 matrix and state-of-the-art random and deterministic sensing matrices

It is noted from Fig. 17 that the proposed sym9 wavelet based sensing matrix clearly outperforms the state-of-the-art random sensing matrices such as Gaussian, Uniform, Toeplitz, Circulant and Hadamard sensing matrices as well as the deterministic DCT and sparse binary sensing matrices in terms of signal reconstruction time. It can be observed from Figs. 18 and 19 that the proposed sym9 wavelet based sensing matrix demonstrates a close comparable performance compared to the state-of-the-art random and deterministic sensing matrices.

#### The overall remark

Thus, it is evident from Figs. 17, 18 and 19 (Tables 28, 29, 30, 31, 32, 33, 34) that the proposed sym9 wavelet based sensing matrix exhibits the better performance compared to the state-of-the-art random and deterministic sensing matrices.

### Subjective quality evaluation

Simple quality measures like SNR do not provide an accurate measure of the speech quality. Hence, speech quality assessment is performed by highly robust and accurate measures such as the mean opinion score (MOS) and perceptual evaluation of speech quality (PESQ) recommended by International Telecommunication Union Telephony (ITU-T) standards.

In this section, the performance of the proposed sensing matrices is evaluated using mean opinion score (MOS). The MOS is a subjective listening test to perceive the speech quality and one of the widely recommended method by ITU standard (ITU-T P.800) (ITU-T 1996).

Table 35 presents subjective evaluation of the reconstructed speech quality using the mean opinion score (MOS) test. The MOS test is performed on a group of seven male listeners and three female listeners. The listeners are required to train and evaluate the quality of the reconstructed speech signal with respect to the original signal. The speech quality is evaluated by rating to a signal within the range of 1–5. The MOS is computed by taking the average score of all the individual listeners and it ranges between 1 (bad speech quality) and 5 (excellent speech quality).

**Table 35:** Subjective evaluation of speech quality using Mean Opinion Score (MOS) test

Sr. no.	Sensing matrix	Listeners										MOS Score
		Male listener							Female listener			
		1	2	3	4	5	6	7	8	9	10	
1.	db1	4	3	3	3	3	3	3	3	4	3	3.2
2.	db2	5	5	4	4	4	4	4	4	3	3	4.0
3.	db3	4	4	4	3	4	4	3	4	4	3	3.7
4.	db4	4	3	3	5	4	4	5	3	3	4	3.8
5.	db5	3	3	3	4	4	4	4	4	3	3	3.5
6.	db6	4	4	4	4	4	4	3	3	4	4	3.8
7.	db7	4	5	3	3	4	4	4	3	4	4	3.8
8.	db8	4	4	4	4	4	4	3	4	4	4	3.9
9.	db9	3	5	3	3	5	4	4	4	3	4	3.8
10.	db10	3	3	4	4	4	4	4	3	4	3	3.6
11.	coif1	4	5	4	5	3	3	4	3	4	4	3.9
12.	coif2	4	3	5	4	3	3	4	3	4	4	3.7
13.	coif3	3	3	3	3	3	3	4	4	3	4	3.3
14.	coif4	4	5	4	4	3	3	4	4	3	4	3.8
15.	coif5	4	5	3	3	3	4	5	4	5	4	4.0
16.	sym4	5	4	4	4	3	3	5	3	3	4	3.8
17.	sym5	5	4	4	4	4	3	4	3	3	4	3.8
18.	sym6	5	5	3	5	4	4	4	3	4	4	4.1
19.	sym7	4	3	4	3	4	4	5	4	3	4	3.8
20.	sym8	4	4	4	4	4	4	5	4	4	4	4.1
21.	sym9	5	5	5	5	5	4	5	4	3	3	4.4
22.	sym10	4	4	4	4	5	4	4	4	4	4	4.1
23.	Battle1	5	5	5	3	5	3	4	3	4	4	4.1
24.	Battle3	4	4	3	3	5	4	3	4	4	4	4.1
25.	Battle5	3	4	4	4	4	4	3	4	4	4	3.8
26.	Beylkin	3	3	4	3	3	3	3	3	4	4	3.3
27.	Vaidynathan	4	3	4	3	4	3	4	3	3	4	3.5
28.	Sparse Binary	4	3	3	3	4	4	5	3	3	3	3.5
29.	DCT matrix	4	5	4	4	5	4	4	4	4	4	4.2
30.	Random Gaussian	4	4	3	4	4	4	3	4	4	4	3.8
31.	Random uniform	3	4	4	3	4	3	4	3	4	4	3.6
32.	Random Toeplitz	4	4	3	3	4	3	4	3	4	4	3.6
33.	Random Circulant	4	4	4	3	4	4	4	4	4	4	3.9
34.	Random Hadamard	4	5	4	4	4	3	4	4	4	4	4.0
35.	Wavelet compression	3	4	3	3	5	5	5	4	3	4	3.9

The following conclusions can be drawn from Table 35.

1. Overall, the Symlets wavelet family achieves the good MOS scores compared to other proposed as well as state-of-the-art sensing matrices.
2. The highest MOS score of 4.4 is achieved by the sym9 wavelet family followed by the sym6, sym8, sym10, Battle1, Battle3 (MOS = 4.1) and followed by the db2, coif5 (MOS = 4.0) respectively. Thus, these MOS scores can be considered as an acceptable score for speech quality.
3. Moreover, the state-of-the-art DCT sensing matrix (MOS = 4.2) and the random Hadamard sensing matrix (MOS = 4.0) shows the good MOS score compared to other state-of-the-art sensing matrices.

However, MOS test frequently requires a sizeable number of listeners to accomplish stable results, and is also the time-consuming and expensive. Nevertheless, subjective quality measures are still one of the most decisive ways to estimate speech quality.

### Objective quality evaluation

The PESQ is a most modern international ITU-T standard (P.862) (ITU-T 2005) for an automated prediction of speech quality by estimating quality scores ranging from −1 to 4.5. In other way, it estimates the MOS (Mean Opinion Score) from both the clean signal and its distorted signal. A higher quality score signifies the better speech quality. Moreover, since human listeners are not required; PESQ is less expensive, accurate and less time-consuming;

Table 36 presents the different objective speech quality metrics such as the PESQ, log-likelihood ratio (LLR) and weighted spectral slope (WSS) along with the three subjective rating scales namely: signal distortion, noise distortion, and overall quality. The ratings are based on the five-point (1–5) MOS scale (Hu and Loizou 2008).

**Table 36:** Objective evaluation of speech quality using measures such as Perceptual Evaluation of Speech Quality (PESQ), Log-Likelihood Ratio (LLR) and Weighted Spectral Slope (WSS)

Sr. no.	Different sensing matrices	Speech distortion	Background distortion	Overall quality	LLR	WSS	PESQ MOS Score
1.	db1	3.4959	2.3958	2.8940	0.618194	45.751366	2.4060
2.	db2	3.3878	2.3902	2.8228	0.735509	40.165614	2.3436
3.	db3	3.1887	2.2231	2.6572	0.791790	50.476578	2.2632
4.	db4	3.5157	2.4790	2.9531	0.701403	37.954092	2.4644
5.	db5	3.7339	2.5471	3.0838	0.535443	34.465134	2.4909
6.	db6	3.4648	2.3444	2.8207	0.612405	40.395098	2.2646
7.	db7	3.4839	2.3110	2.8473	0.619242	39.438455	2.2937
8.	db8	3.5478	2.3337	2.8925	0.560800	41.502149	2.3307
9.	db9	3.7200	2.5313	3.0680	0.521539	37.389013	2.4879
10.	db10	3.7502	2.5574	3.0842	0.508764	34.772191	2.4771
11.	coif1	3.4799	2.2788	2.8831	0.646498	42.964582	2.3862
12.	coif2	3.5241	2.3374	2.9602	0.705475	36.450763	2.4628
13.	coif3	3.8500	2.5787	3.2063	0.527448	29.371499	2.5938
14.	coif4	3.7495	2.5243	3.1167	0.551742	34.069359	2.5387
15.	coif5	3.7443	2.5377	3.1099	0.551411	34.169205	2.5310
16.	sym4	3.2751	2.1626	2.7240	0.658010	63.799345	2.3770
17.	sym5	3.7144	2.4995	3.0894	0.550669	38.139942	2.5395
18.	sym6	3.6323	2.4225	3.0189	0.603048	36.964962	2.4751
19.	sym7	3.8098	2.5829	3.1993	0.569036	31.505778	2.6300
20.	sym8	3.8135	2.5483	3.1875	0.548968	31.641938	2.6039
21.	sym9	3.8163	2.5878	3.1843	0.534342	32.757831	2.6003
22.	sym10	3.8335	2.6147	3.1985	0.536466	30.625240	2.6006
23.	Battle1	3.4479	2.3080	2.8621	0.664658	44.171851	2.3821
24.	Battle3	3.7297	2.4548	3.0899	0.532391	37.307250	2.5212
25.	Battle5	3.6017	2.4841	2.9674	0.578535	39.038937	2.4135
26.	Beylkin	3.6053	2.4451	2.9289	0.546456	35.907963	2.3180
27.	Vaidynathan	3.8044	2.5202	3.1186	0.461501	35.343833	2.4948
28.	Sparse Binary	3.6393	2.7576	3.0467	0.666517	29.706413	2.4868
29.	DCT matrix	3.7628	2.5854	3.1092	0.518874	34.672901	2.5138
30.	Random Gaussian	2.9737	2.6990	2.6855	1.276865	30.019462	2.4291
31.	Random uniform	3.3255	2.7452	2.8794	0.966803	28.298916	2.4577
32.	Random Toeplitz	2.6847	2.6565	2.5249	1.520660	33.026663	2.4108
33.	Random Circulant	3.8147	2.7854	3.1549	0.529008	28.229330	2.5210
34.	Random Hadamard	3.8147	2.7854	3.1549	0.529008	28.229330	2.4934
35.	Wavelet compression	3.6017	2.4841	2.9674	0.578535	39.038937	2.4135

The following conclusions can be drawn from Table 36.

1. The Symlets wavelet family shows the higher signal distortion rating (rating between: 3–4) indicating the fairly natural speech signal quality compared to other proposed and state-of-the art sensing matrices
2. The db5, db9, db10, coif3, coif4, coif5 and Symlets wavelet families shows the good background distortion rating (between rating: 2–3) indicating noticeable noise, but not intrusive and are close comparable to state-of-the art sensing matrices.
3. The db5, db9, db10, coif3, coif4, coif5 and Symlets wavelet families shows the higher signal quality rating (between rating: 3–4) indicating the good/fair speech quality compared to state-of-the art sensing matrices.
4. Overall, the sym9 and the sym10 wavelet family based sensing matrices exhibits good/fair overall quality (For db9 and db10 ratings are 3.1843 and 3.1985 respectively) compared to other proposed and state-of-the art sensing matrices.
5. In terms of objective measures, the sym9 and the sym10 wavelet family based sensing matrices exhibits the lower values of log-likelihood ratio (LLR) and weighted spectral slope (WSS) metrics, indicating the good speech quality and are close comparable with state-of-the art sensing matrices.
6. Finally, in views of PESQ measure, the sym9 and the sym10 wavelet family based sensing matrices exhibits the higher PESQ scores; PESQ = 2.6003 (sym9) and PESQ = 2.6006 (sym10) respectively, signifying the good/fair speech quality compared to other proposed and state-of-the art sensing matrices.

### Information based evaluation

Entropy (H) is a measure of an average information content of a signal (*x*) and widely used in signal processing applications. It is defined as:

22 $$H(X) = - \sum\limits_{i = 1}^{N} {P(x_{i} )\log } P(x_{i} )$$

where *X* = {*x*_1_, *x*_2_,…,*x*_*N*_} is a set of random variable, *P*(*x*_*i*_) is a probability of random variable *x*_*i*_ and *N* is the length of a signal or possible outcomes. It is obvious that the higher signal entropy reflects more information content or more unpredictability of information content.

Table 37 presents the information based evaluation of speech quality. Furthermore, it also provides insights on the selection of the best basis sensing matrix.

**Table 37:** Information based evaluation of speech quality and selection of the best basis sensing matrices

Sr. no.	Different sensing matrices		Entropy of original speech signal	Entropy of reconstructed speech signal	Entropy of sensing matrix
1.	Daubechies wavelet family	db1	10.2888	11.0000	0.1191
2.		db2	10.2888	11.0000	0.4663
3.		db3	10.2888	11.0000	0.6966
4.		db4	10.2888	11.0000	0.9066
5.		db5	10.2888	11.0000	1.0980
6.		db6	10.2888	11.0000	1.2699
7.		db7	10.2888	11.0000	1.4416
8.		db8	10.2888	11.0000	1.6132
9.		db9	10.2888	11.0000	1.7689
10.		db10	10.2888	11.0000	1.9047
11.	Coiflet wavelet family	coif1	10.2888	11.0000	0.6966
12.		coif2	10.2888	11.0000	1.2699
13.		coif3	10.2888	11.0000	1.7689
14.		coif4	10.2888	11.0000	2.1759
15.		coif5	10.2888	11.0000	2.5818
16.	Symmlet wavelet family	sym4	10.2888	11.0000	0.9066
17.		sym5	10.2888	11.0000	1.0980
18.		sym6	10.2888	11.0000	1.2699
19.		sym7	10.2888	11.0000	1.4416
20.		sym8	10.2888	11.0000	1.6132
21.		sym9	10.2888	11.0000	1.7689
22.		sym10	10.2888	11.0000	1.9047
23.	Battle wavelet family	Battle1	10.2888	11.0000	2.0789
24.		Battle3	10.2888	11.0000	3.1632
25.		Battle5	10.2888	11.0000	4.0745
26.	Other wavelet families	Beylkin	10.2888	11.0000	1.7689
27.		Vaidynathan	10.2888	11.0000	2.1759
28.	Random sensing matrices	Random Gaussian	10.2888	11.0000	21.0000
29.		Random uniform	10.2888	11.0000	21.0000
30.		Random Toeplitz	10.2888	11.0000	20.7505
31.		Random Circulant	10.2888	11.0000	11
32.		Random Hadamard	10.2888	11.0000	1
33.	Deterministic sensing matrices	DCT matrix	10.2888	11.0000	19.1415
34.		Sparse Binary	10.2888	11.0000	0.0659
35.	Classical approach	Wavelet compression	10.2888	9.7573	–

The following observations are evident from Table 37.

1. CS based sensing matrices, including proposed as well as state-of-the-art sensing matrices has the higher entropy (H = 11.0) compared to classical wavelet compression technique (H = 9.7573).
2. It is also evident that for the proposed sensing matrices the entropy of the reconstructed speech signal (H = 11.0) is very close to the original signal entropy (H = 10.2888).
3. Furthermore, we have computed the entropy of sensing matrices which shows that state-of-the-art random matrices like Gaussian, Uniform, Toeplitz, Circulant attains higher entropy due to its randomness, followed by deterministic DCT matrix.
4. The proposed sensing matrices such as the Battle (for Battle5, H = 4.0745) and the Symlets wavelet families (for sym9 and sym10, H = 1.7689 and H = 1.9047, respectively) shows the higher entropy compared to the sparse binary (H = 0.0659) and the random Hadamard sensing matrices (H = 1).

### Spectrographic analysis

**Fig. 20:**
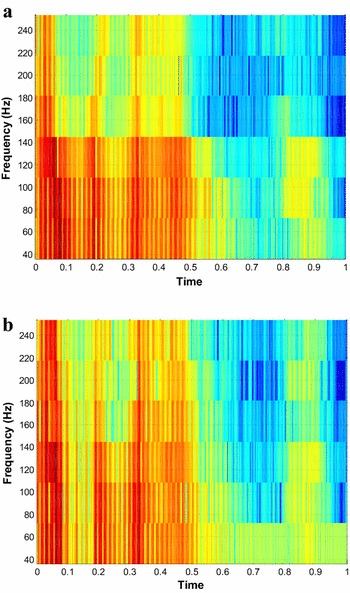
Spectrographic analysis of original and reconstructed speech signal for the proposed sym9 wavelet based sensing matrix (For CR = 0.5). **a** Spectrogram of original speech signal and **b** spectrogram of reconstructed speech signal

The spectrograms are used to visually investigate the joint time–frequency properties of speech signals with intensity or color representing the relative energy of contributing frequencies and it plays an important role in decoding the underlying linguistic massage. Figure 20 shows the spectrographic analysis of the original and the reconstructed speech signal for the proposed sym9 wavelet based sensing matrix (for CR = 0.5). Figure 20a shows the spectrogram of the original input speech signal and Fig. 20b shows the spectrogram of the reconstructed speech signal.

Thus, the spectrographic analysis from Fig. 20 shows that the time–frequency characteristic of the reconstructed spectrogram is a very close to the original speech spectrogram, preserving most of the signal energy. Moreover, the red color shows energy at the highest frequency followed by the yellow, blue respectively, and the white area shows the absence of frequency components.

**Fig. 21:**
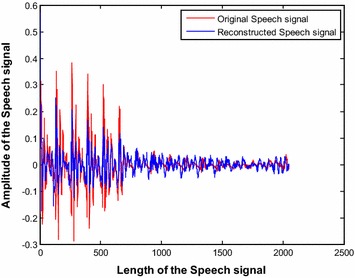
Original and reconstructed speech signal for proposed sym9 wavelet based sensing matrix (for CR = 0.5)

Furthermore, Fig. 21 shows the original and the reconstructed speech signal with the DCT basis for CR = 0.5 (*N* = 2048 and *m* = 1024). It can be observed that the original speech signal is successfully reconstructed using the proposed sym9 wavelet based sensing matrix.

## Conclusions

In this study, an attempt was made to investigate the DWT based sensing matrices for the speech signal compression. This study presents the performance comparison of the different DWT based sensing matrices such as the: Daubechies, Coiflets, Symlets, Battle, Beylkin and Vaidyanathan wavelet families. Further study presents the performance analysis of the proposed DWT based sensing matrices with state-of-the-art random and deterministic sensing matrices. The speech quality is evaluated using subjective and objective measures. The subjective evaluation of speech quality is performed by mean opinion sore (MOS). Moreover, the objective speech quality is evaluated using the PESQ and other measures such as the log-likelihood ratio (LLR) and weighted spectral slope (WSS). Besides, an attempt was made to evaluate the speech quality using the information based measure such as Shannon entropy. In addition, efforts are made to present an insight on the selection of the best basis sensing matrix using the information based measure.

The following major conclusions are drawn based on the investigation:

- Overall, the db10 wavelet based sensing matrix shows the good balance between signal reconstruction error and signal reconstruction time compared to other Daubechies wavelet based sensing matrices. Moreover, the db9 also shows close performance to the db10 and may be the second best choice.
- The coif5 wavelet based sensing matrix shows the good performance, since it requires less reconstruction time, minimum relative error and the high SNR compared to other Coiflets wavelet based sensing matrices. In addition, the coif4 may be the second choice of sensing matrix.
- Overall, the sym9 wavelet sensing matrix demonstrates the less reconstruction time and the less relative error, and thus exhibits the good performance compared to other Symlets wavelet based sensing matrices. Moreover, the sym10 may be the second choice of sensing matrix followed by the sym9.
- The Beylkin wavelet sensing matrix demonstrates the less reconstruction time and relative error, and thus exhibits the good performance compared to the Battle and the Vaidyanathan wavelet based sensing matrices. However, the Battle5 shows a close performance and may be the second best choice of sensing matrix.
- When compared for the best of the DWT sensing matrix, the sym9 wavelet based sensing matrix shows the superior performance compared to the db10, coif5 and Beylkin wavelet based sensing matrices, in the views of signal reconstruction time and relative error. Furthermore, the db10 may be the second best choice of sensing matrix.
- Finally, it is revealed that the proposed sym9 wavelet based sensing matrix exhibits the better performance compared to state-of-the-art random and deterministic sensing matrices in terms of signal reconstruction time and reconstruction error.
- Overall, the Symlets wavelet family achieves good MOS scores compared to other proposed as well as state-of-the-art sensing matrices.
- The highest MOS score of 4.4 is achieved by the sym9 wavelet family followed by the sym6, sym8, sym10, Battle1, Battle3 (MOS = 4.1) and followed by the db2, coif5 (MOS = 4.0) respectively. Thus, these MOS scores can be considered as an acceptable score for speech quality.
- In terms of the PESQ measure, the sym9 and the sym10 wavelet family based sensing matrices exhibits the higher PESQ scores i.e. PESQ = 2.6003 (sym9) and PESQ = 2.6006 (sym10) respectively; signifying the good/fair speech quality compared to other proposed and state-of-the art sensing matrices.
- The sym9 and the sym10 wavelet family based sensing matrices exhibits the lower values of Log-Likelihood Ratio (LLR) and Weighted Spectral Slope (WSS) metrics indicating the good speech quality, and are the close comparable with state-of-the art sensing matrices.
- In views of information based evaluation, CS based sensing matrices, including the proposed DWT based as well as state-of-the-art sensing matrices, has the higher entropy (H = 11.0) compared to the classical wavelet compression technique (H = 9.7573).
- The proposed sensing matrices such as the Battle (For the Battle5, H = 4.0745) and the Symlets wavelet families (For the sym9 and the sym10, H = 1.7689 and H = 1.9047 respectively) shows the higher entropy compared to the sparse binary (H = 0.0659) and the random Hadamard sensing matrices (H = 1).
- Finally, the DWT based sensing matrices exhibits the good promise for speech signal compression.

Thus, this study shows the effectiveness of the DWT based sensing matrices for speech signal processing applications. The scope of this study can be further expanded by investigating the use of the DWT based sensing matrices in other application areas such as music signal processing, under water acoustics and the biomedical signal processing such as the ECG and EEG analysis.

## Abbreviations

DWT: discrete wavelet transform
CS: compressed sensing
CR: compression ratio
RMSE: root mean square error
SNR: signal to noise ratio
MOS: mean opinion score
PESQ: perceptual evaluation of speech quality
